# Supplementary material for: Selective retention of virus-specific tissue-resident T cells in healed skin after recovery from herpes zoster
Source: Nat Commun. 2022 Nov 15;13:6957. doi: 10.1038/s41467-022-34698-4 (PMC9663441; doi:10.1038/s41467-022-34698-4)
Supplement: Supplementary file 1 — Supplementary Information [file 41467_2022_34698_MOESM1_ESM.pdf]

Supplementary Fig. 1, Laing et al.

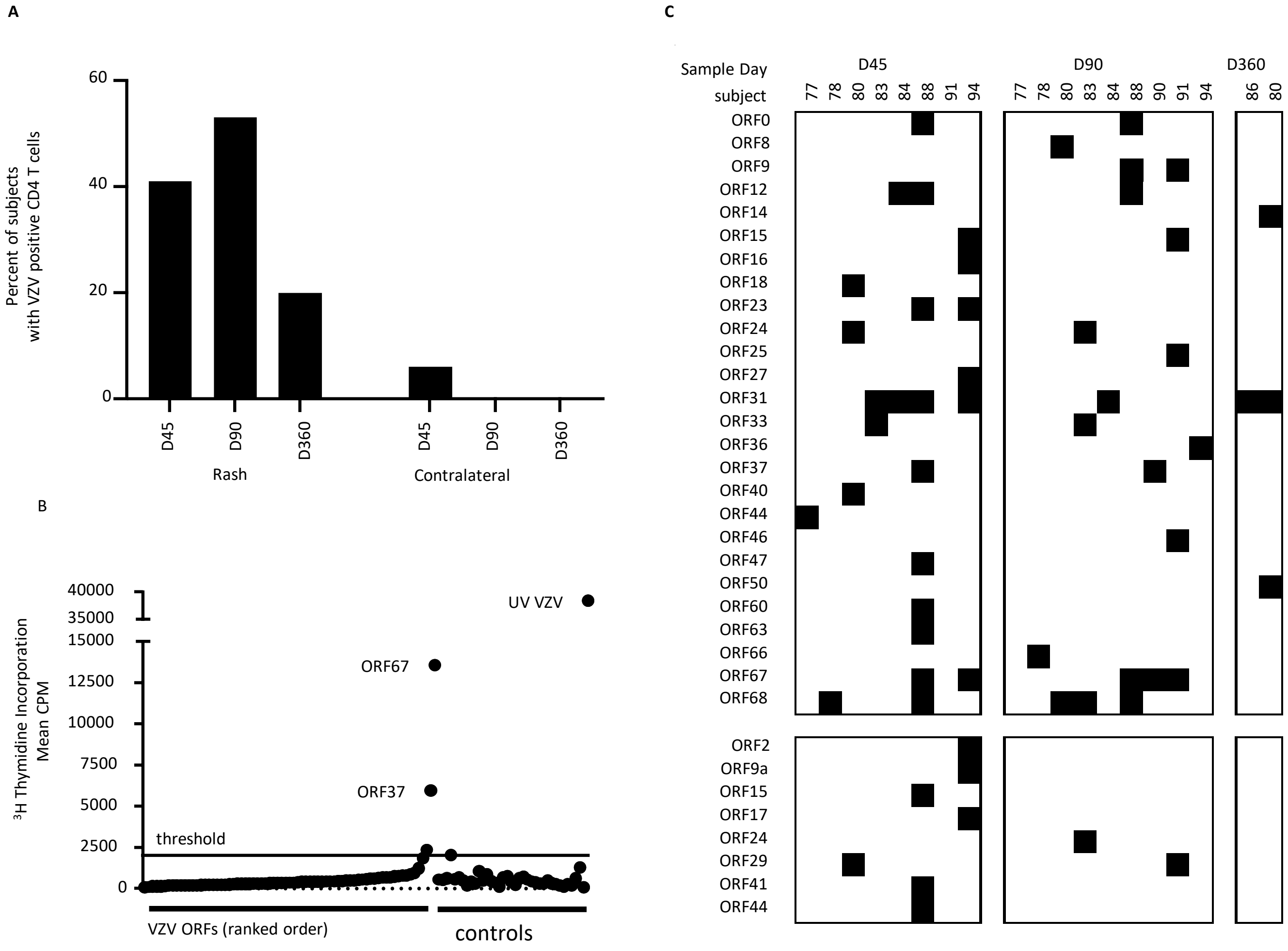

Supplementary Fig. 1 A. Expanded T cell lines from rash and unaffected contralateral skin were scored positive if net CD4 T cell responses to VZV detected by ICS for IFN-γ and/or IL-2 exceeded 3% of all CD4 T cells. Bar graphs show the percent of subjects with positive response on an indicated day (day 45 and day 90 n=17; day 360, n=10). B. Representative screen for T cell proliferative responses to all VZV proteins using 3H-thymidine incorporation. A cell line-specific threshold for positivity was determined using outlier analyses based on the median + (3.2 x MAD) responses of a series of negative controls as detailed in Methods. C. Heatmap showing positive CD4 T cell responses (black squares) to VZV ORFs for T cell lines from the HZ skin sites (HZ-TCL) or contralateral skin sites (CON-TCL) at the day 45, 90, and 360 time-points. Only ORFs that gave at least one positive response are shown. ICS, intracellular cytokine staining; ORF, open reading frame; D45, day 45; D90, day 90; D360, day 360; CPM, counts per minute; VZV, varicella zoster virus; UV VZV, UV-inactivated VZV. Source data are provided as a Source Data file.

Supplementary Fig. 2, Laing et al.

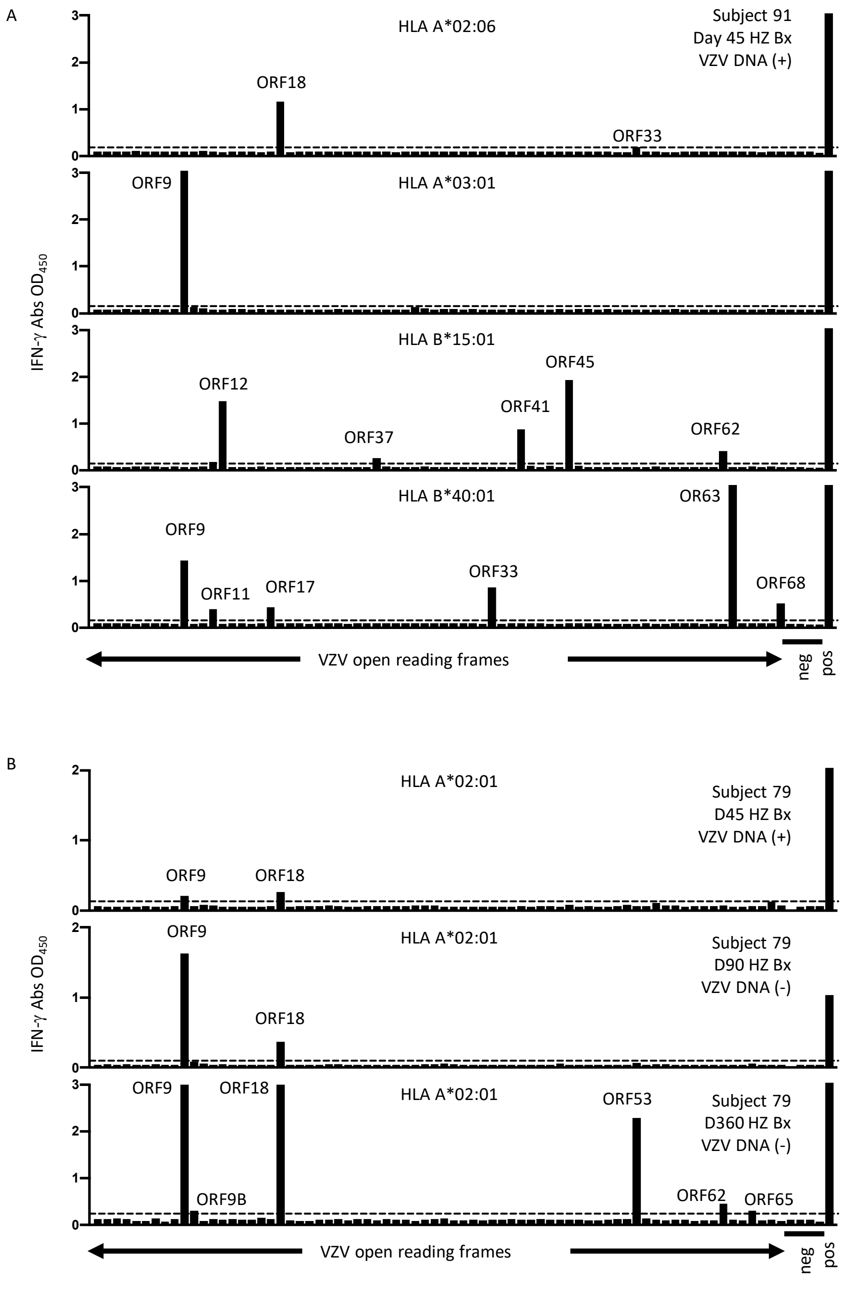

Supplementary Fig. 2. Representative genome-wide screens for CD8 T cell responses to each VZV protein using Cos7-derived artificial antigen presenting cells (aAPC) co-transfected separately with each subject-specific class I HLA and a single VZV ORF and tested in duplicate with biopsy-derived bulk T cells. Negative controls include Cos-7 transfected with empty vector, the indicated HLA cDNA alone, or no transfection. Positive control is unmanipulated Cos-7 with PHA included in assay wells. CD8 T cell activation was detected by IFN-γ ELISA. Bars represent mean of duplicates. Responses were considered positive if they were at least 2X the negative control containing no transfected VZV ORF and had an OD_{450} reading at least 0.15 (horizontal line). **A.** Polyclonal T cells expanded from biopsy 45 days after rash onset from subject 91 show broadly HLA class I-restricted recognition of VZV ORFs. Reactive VZV ORFs are labeled. **B.** HLA-A*02:01 restricted CD8 T cell responses over one year measured in biopsies from subject 79. ORF, open reading frame; D45, day 45; D90, day 90; D360, day 360; HZ Bx, herpes zoster rash site skin biopsy; Abs OD_{450}, absorbance optical density at 450 nanometers; VZV, varicella zoster virus; neg, negative control; pos, positive control. Source data are provided as a Source Data file.

Supplementary Fig. 3, Laing et al.

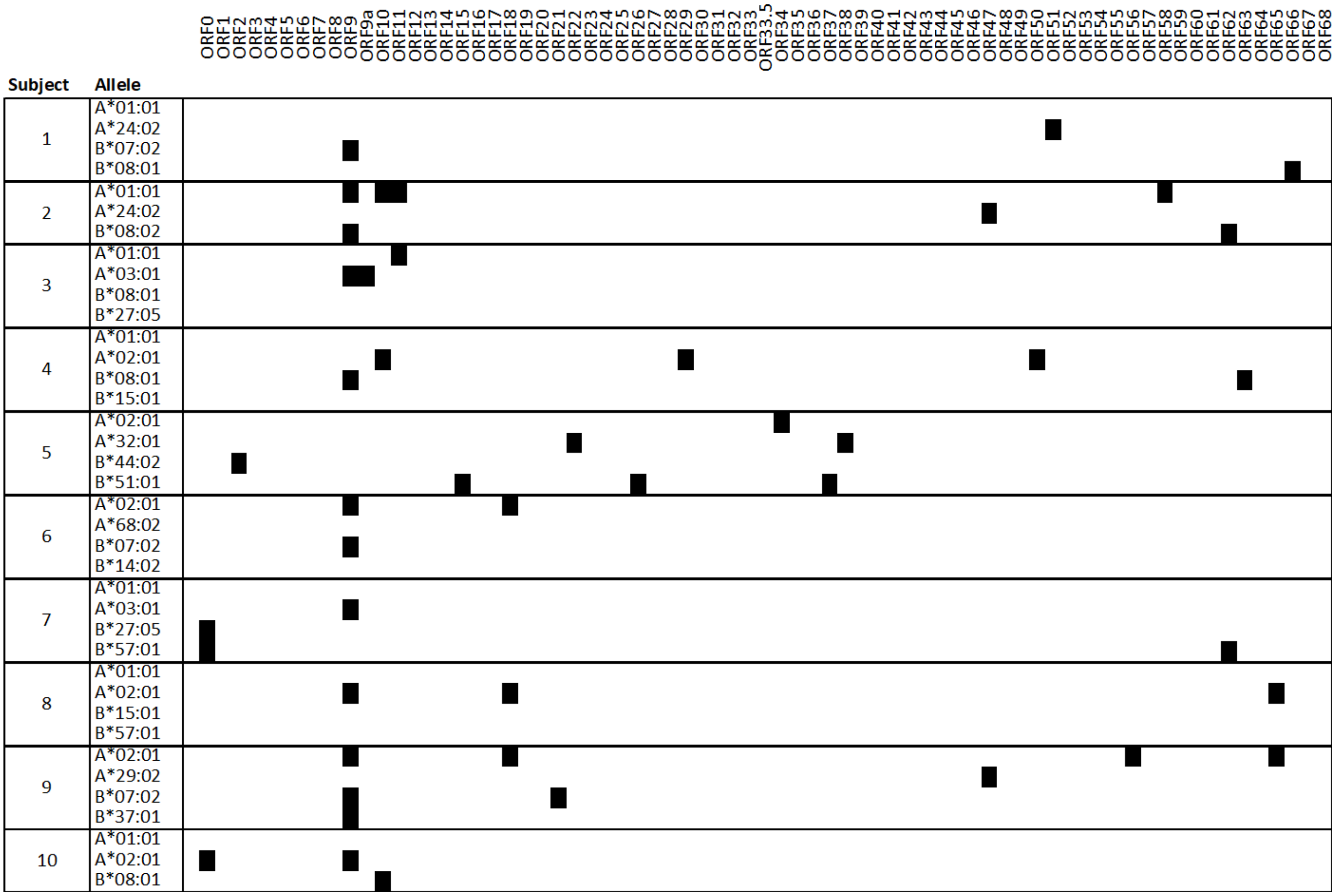

Supplementary Fig. 3. VZV ORFeome responses in blood-derived CD8 T cells from 10 healthy blood donors showed immunoprevalence of ORF9 as a CD8 T cell antigen with diverse HLA-restrictions. Black boxes represent a positive T cell response detected using IFN-γ ELISA for a given subject's CD8 T-cell line following exposure of artificial antigen presenting cells expressing an indicated HLA and VZV ORF. ORF, open reading frame; D45, VZV, varicella zoster virus. Source data are provided as a Source Data file.

Supplementary Figure 4 Laing et al.

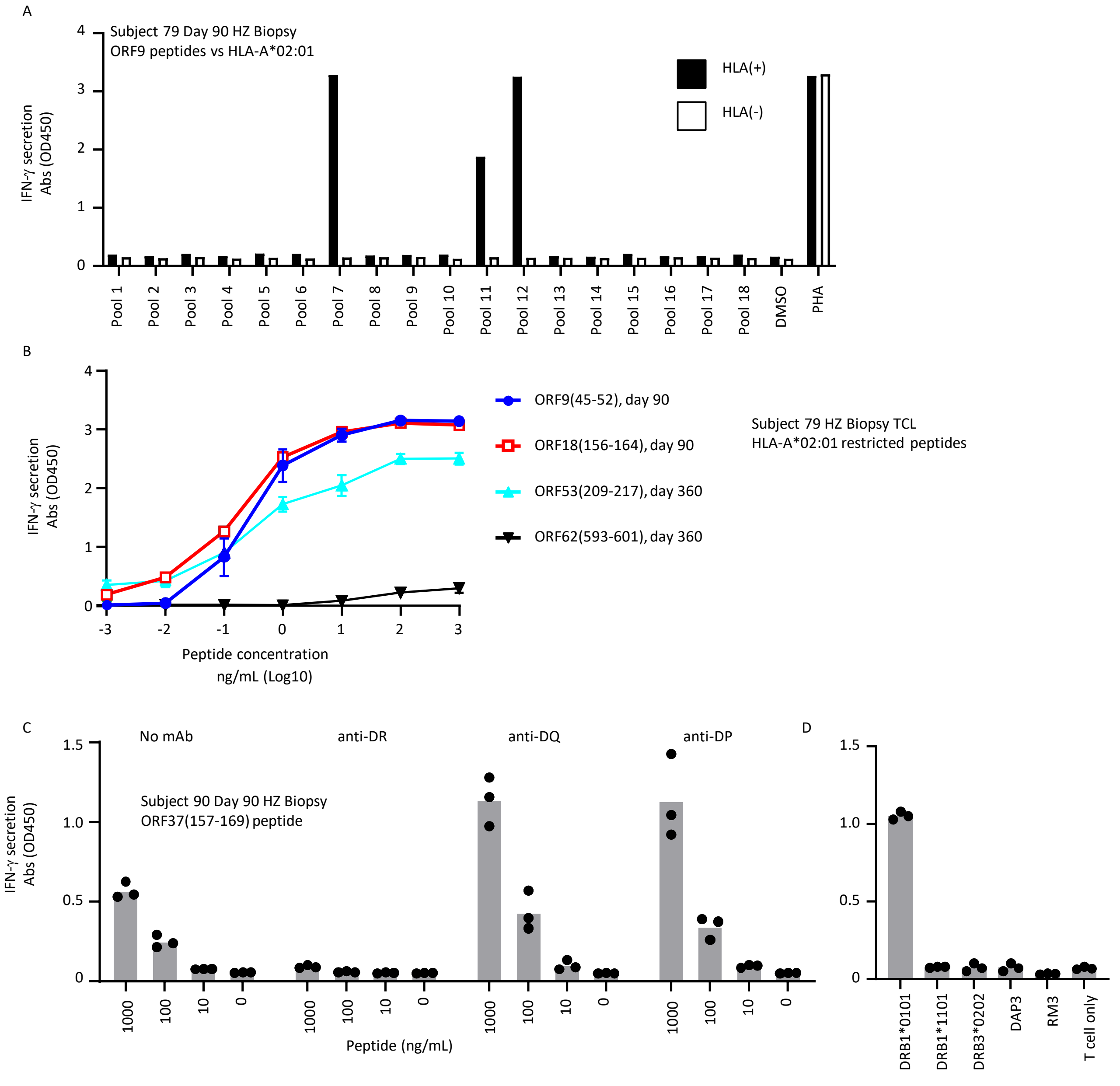

Supplementary Fig. 4: Full-length ORFs that gave positive CD8 or CD4 T cell responses were decoded using peptide pools followed by single peptides. A. The representative example shows VZV ORF9 responses in subject 79 using aAPC expressing (black bars) or lacking (white bars) HLA-A*02:01 to present peptides to skin-derived T cell lines. T cell activation was detected by IFN-γ ELISA. Bars represent mean of duplicate wells. B. Representative dose-response data from subject 79 for four HLA-A*02:01 restricted epitopes using bulk HZ-site expanded T cells. Data represent mean and SD of triplicate wells. C. Example of HLA restriction locus identification for CD4 T cell epitopes using anti-HLA blocking antibodies during T cell assay exposure to the indicated antigenic peptide. B-LCL were used as APC. Assay readout was IFN-γ ELISA. Bars represent mean of triplicate wells. Anti-HLA-DR antibodies are clearly inhibitory for T cell activation by peptide ORF37(157-169) in day 90 HZ biopsy-derived cultures from subject 90. D. Example of HLA allele determination for the using the same cell line/peptide combination described in C. APC express the indicated HLA class II gene or are negative control parental cell lines lacking HLA (DAP.3 and RM3), or T cells alone. Data represent net IFN-γ responses to peptide after subtraction of data from wells exposed to DMSO. Bars show mean of triplicate wells. ORF, open reading frame; VZV, varicella zoster virus; UV VZV, UV-inactivated VZV; Abs OD_{450}, absorbance optical density at 450 nanometers; B-LCL, B-lymphoblastoid cell line; HZ, herpes zoster rash site skin. Source data are provided as a Source Data file.

Supplementary Figure 5 Laing et al.

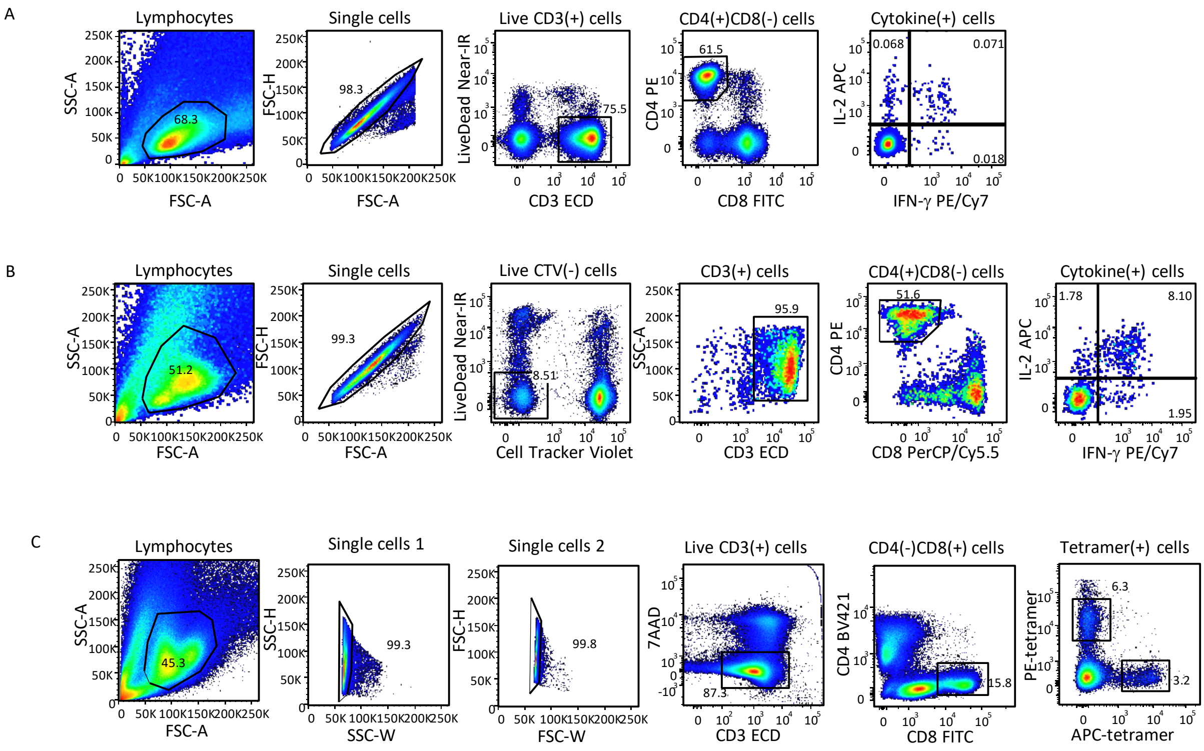

Supplementary Fig 5: Gating strategies for flow cytometric analyses for: A) ex vivo abundance of cytokine (IFN-γ and/or IL-2) positive CD4 T cells in PBMC exposed to VZV or control antigens. Example shows subject 80, day 45 PBMC. B) VZV-specific CD4 T cells in skin biopsy-derived T cell lines when CTV-stained PBMC are used as antigen-presenting cells. Example shows subject 94, day 90 rash site T cell line. C) sorting tetramer-specific CD8 T cells from skin biopsy-derived T cell lines. Example shows subject 79, day 360 rash site T cell line.

Supplementary Table 1. Demographic and serologic characteristics and summary of HZ (shingles, zoster) for study subjects.

Subject	Sex	Herpesvirus serostatus				Zoster episode				HLA-A, -B	
		VZV	HSV-1	HSV-2	CMV	Rash onset to screen, day	Antiviral therapy	Corticosteroid therapy	Dermatome[1]	A	B
77	Male	pos	neg	neg	neg	33	yes	No	L1-L2 (L)	*24:02 *32:01	*07:02 *40:01
78	Female	pos	neg	neg	pos	37	yes	no	T7-T8 (R)	*32:01 *33:03	*15:01 *51:01
79	Female	pos	pos	neg	neg	28	yes	no	T6-T7 (R)	*02:01 *32:01	*15:01
80	Female	pos	pos	neg	neg	31	yes	no	C5-C6 (L)	*03:01	*07:02 *51:01
81	Male	pos	pos	neg	neg	35	yes	no	T4 (R)	*01:01 *02:01	*08:01 *27:05
82	Female	pos	neg	neg	pos	21	yes	no	T6-T7 (L)	*02:01 *29:02	*44:03 *51:01
83	Male	pos	pos	neg	pos	23	yes	no	T8-T9 (R)	*01:01 *30:01	*07:02 *13:02
84	Male	pos	pos	neg	pos	32	yes	no	T7-T8 (R)	*24:02 *33:03	*35:01 *44:03
85	Female	pos	pos	neg	neg	29	yes	no	T4 (R)	*01:01 *26:01	*08:01 *14:01
86	Male	pos	pos	neg	pos	38	yes	no	T6-T7-T8 (L)	*11:01 *30:02	*15:01 *18:01
87	Male	pos	pos	neg	neg	32	yes	no	C7-C8 (L)	*02:01	*44:02
88	Male	pos	neg	neg	neg	30	no	no	T8 (R)	*25:01 *30:01	*13:02 *18:01
89	Male	pos	pos	pos	pos	22	yes	no	T6 (R)	ND	ND
90	Female	pos	neg	neg	neg	31	yes	systemic	T5-T6 (R)	*03:01 *24:02	*35:01 *51:07
91	Male	pos	pos	neg	pos	26	yes	no	T1-T2 (R)	*02:06 *03:01	*15:01 *40:01
92	Male	pos	atypical	neg	pos	31	yes	topical	C2 (L)	*02:01 *02:02	*45:01 *53:01
93	Female	pos	pos	neg	pos	38	yes	no	C8 (R)	*24:02 *29:02	*41:02 *44:03
94	Female	pos	pos	neg	pos	33	yes	systemic	T2 (R)	*02:07 *11:02	*40:01 *46:01

[1] L, T, and C refer to lumbar, thoracic, and cervical dermatomes, respectively. L and R refer to left and right.

Supplementary Table 2. Summary of immune responses in study subjects

	HZ VZV DNA[1] (copies/swab)				CON VZV DNA[2] (copies/swab)				Blood ex vivo ICS[3] (% of CD4 T cells)			HZ skin T cell line ICS (% of CD4)			CON skin T cell line ICS (% of CD4)			Number of VZV proteins recognized by CD4 T cells from HZ-TCL[4]			Number of VZV proteins recognized by CD4 T cells from CON-TCL			Number of VZV proteins recognized by CD8 T cells from HZ-TCL			Number of VZV proteins recognized by CD8 T cells from CON-TCL		
Subject	D30	D45	D90	D360	D30	D45	D90	D360	D45	D90	D360	D45	D90	D360	D45	D90	D360	D45	D90	D360	D45	D90	D360	D45	D90	D360	D45	D90	D360
77	4500	0	0	dec[5]	ns[6]	0	0	dec	0.05	0.05	dec	3.35	3.62	dec	0.06	0.06	dec	0	0	dec	1	0	dec	5	3	dec	0	0	dec
78	0	0	0	0	ns	0	0	257	0.07	0.04	0.06	25.7	14.9	0.09	2.7	0	0	1	1	nd[7]	0	0	nd	1	1	0	0	0	0
79	11250	1953.5	0	0	ns	0	0	0	0.07	0.04	0.06	0.11	0	0.26	0	0	0	nd	nd	nd	nd	nd	nd	2	2	6	1	1	1
80	0	0	0	0	ns	0	0	0	0.13	0.13	0.03	0.36	26.1	13.8	0.2	0.14	0	3	2	3	1	0	0	4	4	0	0	0	0
81	1850	0	0	0	ns	0	0	0	0.06	0.02	0.02	0.09	0.63	0.11	0	0	0	nd	nd	nd	nd	nd	nd	0	0	0	1	1	0
82	900	389.5	0	dec	ns	0	0	dec	0.14	0.13	dec	0.08	0	dec	0.93	1.54	dec	nd	nd	dec	nd	nd	dec	1	1	dec	1	1	dec
83	24000	0	0	0	ns	0	0	0	0.20	0.18	0.03	21.5	5.9	0	0	0	0	2	3	nd	0	1	nd	8	1	2	1	2	0
84	198.5	360	0	dec	ns	0	0	dec	0.29	0.05	dec	7.02	7.33	dec	0.05	0.37	dec	2	1	dec	0	0	dec	4	2	dec	1	2	dec
85	859.5	22573.5	3745	0	ns	0	0	0	0.07	0.02	0.02	1.2	0.09	0	0	0	0	nd	nd	nd	nd	nd	nd	2	1	1	3	0	0
86	5490	1934	0	0	ns	0	0	0	0.07	0.02	0.04	0.73	0.09	4.05	1.38	0	0	nd	nd	nd	nd	nd	nd	4	1	0	0	0	1
87	1650000	84547	181.5	dec	ns	0	0	dec	0.22	0.04	dec	0	0	dec	0.12	0	dec	nd	nd	dec	nd	nd	dec	4	2	dec	0	1	dec
88	0	0	0	dec	ns	0	0	dec	0.17	0.08	dec	43.4	50.9	dec	4.29	0	dec	10	5	dec	3	0	dec	4	0	dec	0	0	dec
89	7000	dec	dec	dec	ns	dec	dec	dec	0.19	0.12	dec	dec	dec	dec	dec	dec	dec	dec	dec	dec	dec	dec	dec	dec	dec	dec	dec	dec	dec
90	33645.5	151.5	0	0	ns	0	0	0	0.16	0.06	0.08	0.47	22.2	0	0	2.89	0	nd	0	nd	nd	2	nd	4	2	0	1	1	0
91	157700	1931.5	0	0	ns	0	0	0	0.45	0.15	0.12	6.11	28.7	0	0	0.32	0	0	1	nd	0	6	nd	13	0	1	0	3	0
92	0	0	0	dec	ns	0	0	dec	0.03	0.15	dec	0	0.01	dec	0.01	0	dec	nd	nd	dec	nd	nd	dec	0	0	dec	0	0	dec
93	1176	0	0	0	ns	0	0	0	0.40	0.09	0.12	1.09	2.57	0.25	0.02	0	0.24	nd	nd	nd	nd	nd	nd	2	3	1	0	1	0
94	0	0	0	0	ns	0	0	0	0.26	0.30	0.14	73.1	9.54	0	0	0	0	6	1	nd	3	0	nd	4	2	3	1	0	0

[1] *HZ VZV DNA, copies of VZV DNA determined by PCR from healed site of herpes zoster eruption*

[2] *CON VZV DNA, copies of VZV DNA determined by PCR from contralateral, uninvolved skin*

[3] *ICS values represent net % of cytokine positive CD4 T cells in whole VZV antigen-exposed samples after subtraction of mock-exposed sample values*

[4] *TCL, T cell line*

[5] *dec, no sample due to subject declining biopsy*

[6] *ns, no swab acquired. A VZV swab sample was not collected from contralateral skin at day 30.*

[7] *nd, not determined due to lack of adequate sample*

Supplementary Table 3. Absorbance OD450 values for anti-VZV gP IgG from ELISA

Specimen		Serum dilution					
Subject	Day	20	80	320	1280	5120	20480
77	45	4.68	4.05	4.01	2.43	1.01	0.34
	60	5.94	4.33	3.43	1.69	0.67	0.22
	90	4.48	4.00	2.89	1.39	0.52	0.16
78	45	5.94	5.63	3.84	2.11	0.81	0.25
	60	5.34	4.14	3.31	1.51	0.53	0.18
	90	3.66	3.90	3.18	1.66	0.66	0.22
	360	3.50	3.67	2.55	1.14	0.41	0.15
79	45	3.60	3.60	2.25	1.09	0.47	0.18
	60	3.62	3.57	2.25	0.91	0.34	0.12
	90	3.61	3.56	2.05	0.83	0.31	0.11
	360	3.62	2.80	1.39	0.49	0.18	0.10
80	45	3.53	3.94	3.38	1.95	0.85	0.40
	60	3.60	3.96	3.07	1.58	0.67	0.26
	90	4.90	4.44	3.25	1.51	0.61	0.27
	360	4.27	3.27	1.89	0.75	0.29	0.15
	500	3.95	3.05	1.72	0.68	0.24	0.11
81	45	4.66	5.93	3.70	1.94	0.78	0.27
	60	4.90	5.93	3.60	1.93	0.76	0.26
	90	5.04	5.93	3.42	1.88	0.84	0.28
	360	5.94	3.98	2.54	1.11	0.37	0.13
82	45	4.11	4.25	4.46	3.22	1.52	0.55
	60	5.92	5.94	5.92	2.32	0.88	0.30
	90	5.92	4.46	3.37	1.76	0.67	0.25
83	45	5.92	5.94	3.83	2.55	0.95	0.34
	60	4.38	5.94	3.83	2.07	0.81	0.26
	90	5.92	5.94	3.62	1.74	0.74	0.26
	360	5.92	4.46	2.83	1.27	0.46	0.17
84	45	5.92	5.94	2.73	1.04	0.37	0.15
	90	5.31	3.93	2.24	0.92	0.31	0.11
85	45	5.94	5.93	3.61	1.74	0.69	0.24
	60	4.98	3.95	2.92	1.34	0.49	0.19
	90	4.04	3.42	2.14	0.95	0.35	0.13
	360	3.68	2.45	1.38	0.64	0.18	0.09
86	45	4.38	4.28	4.11	3.10	1.57	0.57
	60	5.94	5.93	5.96	3.06	1.64	0.51
	90	5.94	4.40	5.96	2.95	1.45	0.80
	360	4.58	4.38	3.78	1.87	0.78	0.23
87	45	5.92	5.94	5.92	3.02	1.45	0.50
	60	4.20	4.74	5.92	2.40	1.06	0.38
	90	3.80	4.28	3.64	1.91	0.70	0.24
88	45	4.06	3.79	2.32	0.90	0.31	0.12
	60	3.81	3.67	2.29	0.87	0.30	0.13
	90	4.30	3.77	2.18	0.78	0.27	0.11
89	45	4.47	5.94	3.89	1.72	0.71	0.28
	60	3.97	4.70	3.02	1.40	0.49	0.17
	90	4.44	3.90	2.75	1.11	0.39	0.14
90	45	3.84	3.95	4.16	3.10	1.52	0.66
	60	3.85	3.75	3.87	2.89	1.38	0.47
	90	3.90	3.74	3.75	2.72	1.26	0.44
	360	4.01	3.77	3.22	1.72	0.65	0.23
91	45	3.72	3.85	4.01	3.14	1.48	0.50
	60	3.97	3.86	3.91	2.33	0.85	0.28
	90	3.77	3.77	3.33	1.64	0.54	0.18
	360	3.83	2.89	1.47	0.54	0.19	0.08
92	45	3.78	3.38	1.95	0.81	0.32	0.13
	60	3.97	3.14	1.69	0.73	0.27	0.13
	90	3.64	3.14	1.73	0.68	0.28	0.14
93	45	3.99	4.04	3.78	3.08	1.69	0.75
	60	3.81	3.67	3.74	2.58	1.24	0.46
	90	3.91	3.93	3.65	2.37	0.96	0.37
	360	3.64	3.48	2.45	1.02	0.33	0.08
94	45	4.49	3.19	1.47	0.55	0.22	0.13
	60	3.97	2.94	1.30	0.49	0.22	0.12
	90	3.85	2.31	0.94	0.34	0.15	0.10
	360	3.60	1.88	0.69	0.24	0.12	0.09

Supplementary Table 4. Immunohistochemistry staining of skin biopsies.

Metric	Subject	D45_CON	D45_HZ	D90_CON	D90_HZ	D360_CON	D360_HZ
A. Number of T cells per mm^2	83	28.93	9.4	67.21	83.26		
	77	8.15	292.27	37.85	17.89		
	87	71.25	322.33	49.71	100.89		
	78	64.72	214	146.24	123.24	99.96	112.28
	91	185.3	308.08	109	116.7	85.8	70.2
	84	39.84	147.12	57.58	109.76		
	90	101.43	220.68	112.66	331.22	158.48	80.02
B. Number of CD4 T cells per mm^2	83	10.27	4.39	36.64	50.47		
	77		121.06	28.33	10		
	87	46.18	141.88	37.8	98.82		
	78	173.4	84.89	59.7	25.3	21.28	35.3
	91	125.37	181.58	64.18	125.36	7.14	43.06
	84	20.33	124.85	17.8	80.35		
	90	45.2	81.62	56.93	276.45	25.47	21.5
C. Number of CD8 T cells per mm^2	83	11.32	4.55	14.46	55.22		
	77		39.2	11.66	7.01		
	87	9.98	88.8	13.36	16.26		
	78	76.73	70.28	42.95	34.71	21.28	21.35
	91	36.64	113.91	41.55	36.56	7.14	7.7
	84	6.43	45.67	5.3	17.63		
	90	18.68	86.01	19.08	161.22	25.47	16.14
D. Percent of CD69+ T cells	83			100	90.5		
	77			94.1	84.7		
	87	82.3	94.1	91.3	100		
	78		93.9	77.1	62	86.1	87.5
	91	100	86.1	100	88.2	76.1	
	84	91.6	71.5	82.3	85.9		
	90	89.6	82.5	100	100	100	80.5
E. Percent of CD103+ T cells	83	11.8	9.1	31.7	9.8		
	77		15	18.5	27		
	87	38.9	23.8	4.4	13.55		
	78	13.3	24.7	11		7	12.2
	91	23.5	33.4	12.4	26	18.7	
	84	12.6	30	6.2	12.1		
	90	13.5	31.7	21.4	15.4	15.8	13.2
F. Percent of TIA-1+ T cells	83	21.1	38.8	26.8	16.4		
	77	55	37.9	16.9	33.3		
	87	9.9	74.3	7.1	28.6		
	78	17.3	31.4	17.4	43.1	23.5	28
	91	13.1	34.4	25.9	13.5	40	16.7
	84	14.8	79	8.3	27.8		
	90	14.4	42.4	10.8	61	9.6	19.6
G. Percent of FOXP3+ T cells	83	8.9	0.8	2.2	5.3		
	77		2.7	7.6	4.5		
	87	0.2	1.7	4.7	19		
	78	0	2.7	4.1	9.8	7.2	5.2
	91		10.4	5.7	14.1	0	6.1
	84	17	4.8	8.5	7.6		
	90	3.6	4	9.9	8.1	8.2	4.6

D45, day45; D90, day 90; D360, day 360; HZ, herpes zoster rash skin site; CON, contralateral skin site

Supplementary Table 5. T cell epitopes found in skin biopsies.

							Subject														
T cell subset	ORF	PEPTIDE NAME	SEQUENCE[1]	IEDB Submission ID	IEDB Epitope ID	HLA	77	78	79	80	81	82	83	84	85	86	87	90	91	93	94
CD8	ORF4	VZV-ORF4(209-221)	VR**HLDETIEKL**VV	1000860	6047223	HLA-A*02:07															■
	ORF9	VZV-ORF9(41-53)	DSDDSLGYITTVG	1000838	4524292	HLA-A*02:01			■												
		VZV-ORF9(45-57)	SLGYITTVGADSP	1000838	4524324	HLA-A*02:01			■		■						■				
		VZV-ORF9(97-109)	AF**REARLRHEL**VE	1000860	6047197	HLA-B*40:01															■
		VZV-ORF9(109-121)	ED**AVYENPLSVEK**	1000838	4524293	HLA-A*03:01				■								■	■		
		VZV-ORF9(109-121)	EDAVYENPLSVEK	1000838	4524293	HLA-A*11:01										■					
		VZV-ORF9(109-121)	EDAVYENPLSVEK	1000838	4524293	HLA-A*11:02															■
		VZV-ORF9(157-169)	SFSTAPKTATSSW	1000838	4524322	HLA-B*07:02	■			■			■								
		VZV-ORF9(209-221)	NAELDRLLTGAVI	1000838	4524312	HLA-B*14:01									■						
		VZV-ORF9(213-225)	DRLLTGAVIRITV	1000838	4524291	HLA-B*14:01									■						
		VZV-ORF9(277-289)	RTDTQTTGRITNR	1000838	4524321	HLA-A*33:03		■						■							
		VZV-ORF9(281-293)	QTTGRITNRSRAR	1000838	4524317	HLA-A*33:03		■						■							
	ORF11	VZV-ORF11(666-674)	ETDLMVSAY	1000860	6047205	HLA-A*01:01							■								
	ORF12	VZV-ORF12(17-29)	**KSYDGSYQSF**NAG	1000860	6047214	HLA-B*15:01													■		
	ORF18	VZV-ORF18(153-165)	ILM**ILIEGIFFV**S	1000838	4524302	HLA-A*02:01			■			■					■				
		VZV-ORF18(153-165)	ILMILIEGIFFVS	1000838	4524302	HLA-A*02:06													■		
		VZV-ORF18(156-164)	ILIEGIFFV	1000838	4524300	HLA-B*13:02							■								
		VZV-ORF18(157-169)	LIEGIFFVSSFAA	1000838	4524309	HLA-A*02:01			■												
	ORF20	VZV-ORF20(249-258)	HVFPHRFISF	1000838	4524299	HLA-A*32:01	■														
	ORF24	VZV-ORF24(22-31)	RIRLVVPSAL	1000838	4524319	HLA-B*07:02				■											
	ORF31	VZV-ORF31(157-165)	KENIAAYKF	pending	pending	HLA-B*44:03								■							
	ORF34	VZV-ORF34(232-240)	AVLCLYLMY	1000860	6047200	HLA-A*29:02														■	
	ORF36	VZV-ORF36(217-225)	KTNNWHAGW	1000838	4524308	HLA-A*32:01	■														
	ORF38	VZV-ORF38(187-195)	GLFDGTPEI	1000860	6047208	HLA-A*02:01											■				
	ORF41	VZV-ORF41(161-173)	AAG**AQILPHEVL**R	1000860	6047196	HLA-B*15:01													■		
	ORF47	VZV-ORF47(141-149)	GTYGRVHIY	1000860	6047209	HLA-A*29:02														■	
	ORF53	VZV-ORF53(209-217)	FLYEGVLWL	1000860	6047207	HLA-A*02:01			■								■				
	ORF62	VZV-ORF62(593-601)	ALWALPHAA	1000860	6047198	HLA-A*02:01			■												
	ORF63	VZV-ORF63(57-69)	PARLVEDINRVFL	1000838	4524313	HLA-B*40:01	■												■		
	ORF63	VZV-ORF63(61-73)	VEDINRVFLCIAQ	1000838	4524329	HLA-B*40:01	■												■		
	ORF64	VZV-ORF64(107-116)	MPDPQSHLEL	1000860	6047216	HLA-B*07:02							■								
	ORF68	VZV-ORF68(176-184)	EVSVEENHPFTLR	1000860	6047206	HLA-B*40:01													■		
	ORF68	VZV-ORF68(211-219)	DAAPAIQHI	1000860	6047202	HLA-B*51:01				■											
	ORF68	VZV-ORF68(309-321)	TSTYATFLVTWKG	1000838	4524326	HLA-A*24:02	■														
CD4	ORF9	VZV-ORF9(149-161)	AIASGRPISFSTA	1000838	4524284	HLA-DRB1*04:04				■											
		VZV-ORF9(185-197)	VRRVAAMQAQKAA	1000838	4524332	HLA-DQB1*06:02											■				
		VZV-ORF9(185-197)	VRRVAAMQAQKAA	1000838	4524332	HLA-DRB1*04:04				■											
	ORF12	VZV-ORF12(409-421)	ETSTHMWFSRAVA	1000838	4524295	HLA-DR											■				
		VZV-ORF12(413-425)	HMWFSRAVAQSIL	1000838	4524298	HLA-DRB1*07:01											■				
		VZV-ORF12(437-449)	QVLAASKRYTPLH	1000838	4524318	HLA-DR											■				
	ORF29	VZV-ORF29(933-945)	RKDVTSAADIETQ	1000860	6047219	HLA-DQ				■											
	ORF37	VZV-ORF37(117-129)	GRFGFLSHPVTPD	1000838	4524297	HLA-DP											■				
		VZV-ORF37(157-169)	LVWHLERAETAAT	1000860	6047215	HLA-DRB1*01:01												■			
		VZV-ORF37(657-669)	YVLILPAVQGHSY	1000860	6047224	unknown												■			
	ORF41	VZV-ORF41(157-169)	VERTAAGAQILPH	1000838	4524330	HLA-DP											■				
	ORF63	VZV-ORF63(49-61)	CTPGWEIHPARLV	1000838	4524289	HLA-DRB1*07:01											■				
		VZV-ORF63(89-101)	CLDFYLMGRTRQR	1000838	4524288	HLA-DR											■				
	ORF67	VZV-ORF67(46-65)	DNYTEIKGQLVFIGEQLPTG	1000860	6047203	HLA-DRB1*01:01												■			
		VZV-ORF67(91-110)	RYDGCPRIRTSAFISCRYKH	1000860	6047221	unknown											■				
		VZV-ORF67(136-155)	PGINDAGVYVLLVRLDHSRS	1000860	6047217	HLA-DRB1*11:01												■			
		VZV-ORF67(154-173)	RSTDGFILGVNVYTAGSHHN	1000860	6047220	HLA-DR												■			
	ORF68	VZV-ORF68(129-141)	DTGIHVIPTLNGD	1000838	4524332	HLA-DRB1*04:04				■											
		VZV-ORF68(477-489)	VFVVYFNGHVEAV	1000838	4524331	HLA-DRB1*15:01											■				

[1] Bold sections represent epitope regions confirmed with shorter peptides

[2] CD4 T cell epiotpes were on mapped for subjects 80, 87 and 90.

Supplementary Table 6: Productive reads and clonotype diversity for CDR3 sequences calculated using VDJTools CalcDiversityStats function

Specimen type*	Day	Productive TRB reads	Number of unique clonotypes (diversity)
HZ Biopsy	45	56188	14022
HZ Biopsy	90	29070	9684
HZ Biopsy	360	13092	4920
Con Biopsy	45	7318	2891
Con Biopsy	90	11256	4040
Con Biopsy	360	21964	6580
PBMC	45	309879	197002
PBMC	90	264437	184989
PBMC	360	329770	226618

* HZ, herpes zoster rash site; Con, contralateral skin site

Supplementary Table 7. *TRB* CDR3 sequences of VZV epitope-specific CD8 T cells.

In column names: FREQ = frequency among all TRB sequences, CON = contralateral, D = day.

ORF	Epitope	cdr3nt	cdr3aa	v	d	j	Rank	FREQ_RASH_D45_SKIN	FREQ_CON_D45_SKIN	FREQ_PBMC_D45	FREQ_RASH_D90_SKIN	FREQ_CON_D90_SKIN	FREQ_PBMC_D90	FREQ_RASH_D360_SKIN	FREQ_CON_D360_SKIN	FREQ_PBMC_D360
ORF9	SLGYITTVG	TGTGCCAGCAGTAAATATACAGGGGACTATGGCTACACCTTC	CASSKYTGDYGYTF	TRBV6-5	TRBD1-1	TRBJ1-2	1	6.52E-02	4.65E-03	3.02E-03	5.83E-02	7.20E-03	1.53E-03	2.50E-02	3.37E-03	1.73E-03
ORF9	SLGYITTVG	TGTGCCAGCAGTTTAAATACGGCAGCGCCGCAAGAGACCCAGTACTTC	CASSLNTAAPQETQYF	TRBV12	TRBD2-1	TRBJ2-5	2	1.06E-02	4.10E-04	2.13E-04	1.04E-02	5.33E-04	6.43E-05	3.28E-03	2.28E-04	7.28E-05
ORF9	SLGYITTVG	TGTGCCAGCAGGCCCGCCTTGGGCGCAAACTATGGCTACACCTTC	CASRPALGANYGYTF	TRBV6-5	TRBD1-1	TRBJ1-2	3	4.84E-03	1.37E-04	3.39E-04	4.27E-03	3.55E-04	1.17E-04	1.22E-03	9.11E-05	2.49E-04
ORF9	SLGYITTVG	TGTGCCAGCAGCACGGGACTGCCGAATGGCTACACCTTC	CASSTGLPNGYTF	TRBV7-8	TRBD2-1	TRBJ1-2	4	3.93E-03	0.00E+00	1.39E-04	3.96E-03	4.44E-04	4.92E-05	1.15E-03	1.37E-04	5.16E-05
ORF9	SLGYITTVG	TGTGCCAGCAGCCCTTTAGGAGCCATGAACACTGAAGCTTTCTTT	CASSPLGAMNTEAFF	TRBV7-2	TRBD2-1	TRBJ1-1	5	3.36E-03	0.00E+00	1.74E-04	3.34E-03	3.55E-04	4.92E-05	1.30E-03	4.55E-05	7.58E-05
ORF9	SLGYITTVG	TGTGCCAGCAGCTTGGGGAAACAGGGAGACTCACCCCTCCACTTT	CASSLGKQGDSPLHF	TRBV7-6	TRBD1-1	TRBJ1-6	6	2.74E-03	1.37E-04	3.87E-05	2.30E-03	0.00E+00	2.27E-05	9.93E-04	0.00E+00	1.82E-05
ORF9	SLGYITTVG	TGTGCCATCAGTGAGCCGGCGCCGGGACTAGCGGGAGTAGTGAGCACAGATACGCAGTATTTT	CAISEPAPGLAGVVSTDTQYF	TRBV10-3	TRBD2-1	TRBJ2-3	7	9.61E-04	2.73E-04	4.20E-05	7.57E-04	8.88E-05	1.51E-05	3.82E-04	4.55E-05	1.52E-05
ORF9	SLGYITTVG	TGTGCCATCAGTGAGTCGCAGACGGCCTCCTCTGGGGAGACCCAGTACTTC	CAISESQTASSGETQYF	TRBV10-3	TRBD1	TRBJ2-5	8	4.45E-04	2.73E-04	4.20E-05	5.16E-04	8.88E-05	1.89E-05	2.29E-04	4.55E-05	1.82E-05
ORF9	SLGYITTVG	TGTGCCAGCAGCTCCCGCGTGGGGCAGGACCACGAGCAGTACTTC	CASSSRVGQDHEQYF	TRBV7-6	TRBD1-1	TRBJ2-7	9	2.31E-04	0.00E+00	5.42E-04	5.85E-04	0.00E+00	4.73E-04	1.53E-04	0.00E+00	5.00E-04
ORF9	SLGYITTVG	TGTGCCAGCAGCTTATTCGACAGGGATGGCTACACCTTC	CASSLFDRDGYTF	TRBV7-9	TRBD1-1	TRBJ1-2	10	2.14E-04	1.37E-04	2.29E-04	6.19E-04	8.88E-05	2.76E-04	2.29E-04	4.55E-05	2.64E-04
ORF9	SLGYITTVG	TGTGCCAGCAGCCAGATAGCGGGAGATCGATCCAGATACGCAGTATTTTGG	CASSQIAGDRSRYAVFW	TRBV11-3	TRBD2-1	TRBJ2-3	11	1.78E-04	2.32E-03	0.00E+00	3.78E-04	1.87E-03	0.00E+00	1.83E-03	1.41E-03	0.00E+00
ORF9	SLGYITTVG	TGTGCCAGCAGCACCCCCGGGACAGCGTCCTACGAGCAGTACTTC	CASSTPGTASYEQYF	TRBV7-9	TRBD1-1	TRBJ2-7	12	1.42E-04	0.00E+00	5.33E-03	3.44E-05	0.00E+00	3.68E-03	0.00E+00	4.55E-05	4.98E-03
ORF9	SLGYITTVG	TGTGCCAGCAGGGGACACCCCCTAGGAGAGACCCAGTACTTC	CASRGHPLGETQYF	TRBV28-1	TRBD1-1	TRBJ2-5	13	1.25E-04	0.00E+00	9.68E-06	1.38E-04	0.00E+00	1.13E-05	0.00E+00	0.00E+00	3.03E-06
ORF9	SLGYITTVG	TGTGCCAGCAGCCCCGGAGCTAATAATTCACCCCTCCACTTT	CASSPGANNSPLHF	TRBV5-5	TRBD2-1	TRBJ1-6	14	1.07E-04	0.00E+00	1.41E-02	1.03E-04	1.78E-04	8.17E-03	1.53E-04	4.55E-05	1.31E-02
ORF9	SLGYITTVG	TGTGCCAGCTCACCTACAGGGAGCTCTGGAAACACCATATATTTT	CASSPTGSSGNTIYF	TRBV18-1	TRBD1-1	TRBJ1-3	15	1.07E-04	6.83E-04	1.61E-05	1.38E-04	1.24E-03	1.51E-05	3.06E-04	1.00E-03	6.06E-06
ORF9	SLGYITTVG	TGTGCCAGCAGTGAAGTGGGCAGCTATGGCTACACCTTC	CASSEVGSYGYTF	TRBV6-1	TRBD1-1	TRBJ1-2	16	8.90E-05	0.00E+00	1.45E-03	6.88E-05	8.88E-05	8.58E-04	0.00E+00	0.00E+00	1.06E-03
ORF9	SLGYITTVG	TGTGCCAGCAGTTACAGTCACACAGATACGCAGTATTTT	CASSYSHTDTQYF	TRBV6-5	TRBD1	TRBJ2-3	17	7.12E-05	4.10E-04	0.00E+00	1.72E-04	5.33E-04	7.56E-06	3.06E-04	5.92E-04	6.06E-06
ORF9	SLGYITTVG	TGTGCCAGCAGCCTACGTATAGTTAGTGAAGCTTTCTTT	CASSLRIVSEAFF	TRBV11-2	TRBD2-1	TRBJ1-1	18	1.78E-05	4.10E-04	1.16E-04	3.44E-05	2.67E-04	9.83E-05	3.06E-04	1.82E-04	8.19E-05
ORF9	SLGYITTVG	TGTGCCAGCAGGGACCCAGGGTTGAGTCAGCCCCAGCATTTT	CASRDPGLSQPQHF	TRBV6	TRBD1-1	TRBJ1-5	19	1.78E-05	0.00E+00	4.20E-05	0.00E+00	0.00E+00	7.56E-06	0.00E+00	0.00E+00	2.12E-05
ORF9	SLGYITTVG	TGTGCCAGCAGGGCAGGGGGCACTGAAGCTTTCTTT	CASRAGGTEAFF	TRBV27-1	TRBD1-1	TRBJ1-1	20	0.00E+00	0.00E+00	0.00E+00	0.00E+00	0.00E+00	0.00E+00	0.00E+00	0.00E+00	0.00E+00
ORF9	SLGYITTVG	TGCGCCAGCAGGGGACAGGGTCGGGACAATGAGCAGTTCTTC	CASRGQGRDNEQFF	TRBV5-1	TRBD1-1	TRBJ2-1	21	0.00E+00	0.00E+00	6.45E-06	3.44E-05	0.00E+00	3.78E-06	0.00E+00	0.00E+00	3.03E-06
ORF9	SLGYITTVG	TGTGCCAGCAGCCGTGGTGAGGGATATGAAGCTTTCTTT	CASSRGEGYEAFF	TRBV11-3	TRBD2-1	TRBJ1-1	22	0.00E+00	0.00E+00	1.94E-05	0.00E+00	0.00E+00	1.89E-05	0.00E+00	0.00E+00	1.52E-05
ORF9	SLGYITTVG	TGTGCCACCAGTGCATCACGTGGTGGTACAGGGGGCGGTTTTTATGGCTACACCTTC	CATSASRGGTGGGFYGYTF	TRBV24-1	TRBD1-1	TRBJ1-2	23	0.00E+00	0.00E+00	3.87E-05	0.00E+00	0.00E+00	1.89E-05	0.00E+00	0.00E+00	4.55E-05
ORF18	ILIEGIFFVS	TGTGCCAGCAGCTTCGGGACAGTGGACCCCTATGGCTACACCTTC	CASSFGTVDPYGYTF	TRBV7-8	TRBD1-1	TRBJ1-2	1	8.46E-02	0.00E+00	4.71E-04	5.58E-02	8.88E-05	2.31E-04	1.13E-02	0.00E+00	2.37E-04
ORF18	ILIEGIFFVS	TGTGCCACCAGCACACGAGAGGCGACAGTCTATGGCTACACCTTC	CATSTREATVYGYTF	TRBV15-1	TRBD1-1	TRBJ1-2	2	6.40E-02	2.73E-04	2.26E-03	3.51E-02	6.22E-04	1.35E-03	6.11E-03	1.32E-03	1.21E-03
ORF18	ILIEGIFFVS	TGTGCCAGCAGTGAAGCGCAGGCCCCAAGCCATGGCTACACCTTC	CASSEAQAPSHGYTF	TRBV6-1	TRBD1-1	TRBJ1-2	3	1.05E-02	0.00E+00	1.45E-04	6.23E-03	0.00E+00	6.81E-05	7.64E-04	0.00E+00	4.25E-05
ORF18	ILIEGIFFVS	TGTGCCAGCAGCCCTCAGGGGGCTCTGAACACTGAAGCTTTCTTT	CASSPQGALNTEAFF	TRBV14-1	TRBD1-1	TRBJ1-1	4	4.66E-03	0.00E+00	3.23E-05	4.09E-03	0.00E+00	1.89E-05	9.17E-04	0.00E+00	2.12E-05
ORF18	ILIEGIFFVS	TGTGCCAGCAGTGACCCTAAAGGGGGTAGCGGGAGGTTGGGGGAGCTGTTTTTT	CASSDPKGGSGRLGELFF	TRBV11-2	TRBD2-1	TRBJ2-2	5	2.90E-03	0.00E+00	2.90E-05	2.27E-03	0.00E+00	1.51E-05	1.07E-03	0.00E+00	1.21E-05
ORF18	ILIEGIFFVS	TGTGCCAGCAGCACGACAGGGGCCCTGGGGACCGGGGAGCTGTTTTTT	CASSTTGALGTGELFF	TRBV13-1	TRBD1-1	TRBJ2-2	6	2.83E-03	4.10E-04	1.74E-04	2.17E-03	1.24E-03	1.10E-04	1.37E-03	5.46E-04	1.67E-04
ORF18	ILIEGIFFVS	TGTGCCAGCAGTTACTTCTTCACAAGGACTGAGCAGTTCTTC	CASSYFFTRTEQFF	TRBV6-6	TRBD1	TRBJ2-1	7	2.67E-03	0.00E+00	3.23E-05	1.82E-03	0.00E+00	2.27E-05	8.40E-04	0.00E+00	2.43E-05
ORF18	ILIEGIFFVS	TGTGCCAGCAGCCAAGGGGGTGCGATGGCCACTGAAGCTTTCTTT	CASSQGGAMATEAFF	TRBV14-1	TRBD1	TRBJ1-1	8	2.62E-03	0.00E+00	0.00E+00	1.79E-03	0.00E+00	3.78E-06	0.00E+00	0.00E+00	0.00E+00
ORF18	ILIEGIFFVS	TGCAGTGCTAGGGACGGGGGCACTGAAGCTTTCTTT	CSARDGGTEAFF	TRBV20	TRBD2-1	TRBJ1-1	9	1.42E-03	0.00E+00	6.45E-06	4.47E-04	0.00E+00	0.00E+00	3.82E-04	0.00E+00	3.03E-06
ORF18	ILIEGIFFVS	TGTGCCAGCATAGGAAGCTACAATGAGCAGTTCTTC	CASIGSYNEQFF	TRBV2-1	TRBD1	TRBJ2-1	10	9.61E-04	0.00E+00	5.81E-05	9.63E-04	8.88E-05	2.65E-05	3.82E-04	1.37E-04	1.52E-05
ORF18	ILIEGIFFVS	TGTGCCAGTAGTATGACTAGTGGGTCCTACAATGAGCAGTTCTTC	CASSMTSGSYNEQFF	TRBV19-1	TRBD2-1	TRBJ2-1	11	7.12E-04	0.00E+00	4.84E-04	1.03E-03	8.88E-05	3.03E-04	2.29E-04	1.37E-04	3.43E-04
ORF18	ILIEGIFFVS	TGTGCCAGCTCACAGGGATCCGCCCCGATCTATGGCTACACCTTC	CASSQGSAPIYGYTF	TRBV19-1	TRBD1-1	TRBJ1-2	12	6.23E-04	0.00E+00	5.29E-04	5.50E-04	8.88E-05	2.80E-04	3.06E-04	9.11E-05	2.97E-04
ORF18	ILIEGIFFVS	TGTGCCTCCAAGCGGGGTTCCTACGAGCAGTACTTC	CASKRGSYEQYF	TRBV28-1	TRBD2-1	TRBJ2-7	13	5.87E-04	1.37E-04	4.20E-04	6.88E-04	8.88E-05	4.58E-04	1.53E-04	4.55E-05	8.13E-04
ORF18	ILIEGIFFVS	TGCGCCAGCAGCCCCCCTGACAGCAATCAGCCCCAGCATTTT	CASSPPDSNQPQHF	TRBV4-1	TRBD1	TRBJ1-5	14	5.87E-04	0.00E+00	0.00E+00	3.44E-04	0.00E+00	0.00E+00	7.64E-05	0.00E+00	0.00E+00
ORF18	ILIEGIFFVS	TGTGCCAGCAGCTTGGGTTGGCGGAGCTCCTATAATTCACCCCTCCACTTT	CASSLGWRSSYNSPLHF	TRBV5-5	TRBD2-1	TRBJ1-6	15	5.52E-04	1.37E-04	9.68E-05	8.94E-04	8.88E-05	1.02E-04	5.35E-04	3.64E-04	1.36E-04
ORF18	ILIEGIFFVS	TGTGCCAGCAGTGACCCTAAAGGGGGTAGCGGGAGGGTGGGGGAGCTGTTTTTT	CASSDPKGGSGRVGELFF	TRBV11-2	TRBD2-1	TRBJ2-2	16	5.52E-04	0.00E+00	6.45E-06	3.10E-04	0.00E+00	3.78E-06	1.53E-04	0.00E+00	9.10E-06
ORF18	ILIEGIFFVS	TGCGCCAGCAGTGATAGAGGTTTGTGGGAGGGAGAGATACGCAGTATTTTG	CASSDRGLWEGEIRSIL	TRBV10-1	TRBD2-1	TRBJ2-3	17	3.56E-04	0.00E+00	3.23E-06	3.78E-04	0.00E+00	3.78E-06	7.64E-05	0.00E+00	6.06E-06
ORF18	ILIEGIFFVS	TGTGCCAGCAGCTTACGGGGGGGCTCCTACGAGCAGTACTTC	CASSLRGGSYEQYF	TRBV7-8	TRBD2-1	TRBJ2-7	18	3.38E-04	2.73E-04	9.65E-03	2.06E-04	1.78E-04	8.91E-03	2.29E-04	0.00E+00	5.92E-03
ORF18	ILIEGIFFVS	TGCGCCAGTGTCCCCAGGGGGCAGGTGGAAACACCATATATTTG	CASVPRGQVETPYIL	TRBV5-1	TRBD1-1	TRBJ1-3	19	3.38E-04	0.00E+00	2.58E-04	5.16E-04	8.88E-05	1.13E-04	3.06E-04	4.55E-05	1.39E-04
ORF18	ILIEGIFFVS	TGTGCCAGTAGTACGACTAGCGGTTCCTACAATGAGCAGTTCTTC	CASSTTSGSYNEQFF	TRBV19-1	TRBD2-1	TRBJ2-1	20	3.38E-04	0.00E+00	1.23E-04	3.78E-04	1.78E-04	1.13E-04	3.06E-04	0.00E+00	1.00E-04
ORF18	ILIEGIFFVS	TGTGCCAGCAGCTCCGAGACAGGGCGCTCCTACGAGCAGTACTTC	CASSSETGRSYEQYF	TRBV9-1	TRBD1-1	TRBJ2-7	21	1.96E-04	2.60E-03	6.45E-06	6.54E-04	2.75E-03	7.56E-06	1.15E-03	7.28E-04	1.21E-05
ORF18	ILIEGIFFVS	TGTGCCAGCAGCGACGGGACTCGCCACGGGGGTTACCAAGAGACCCAGTACTTC	CASSDGTRHGGYQETQYF	TRBV7-8	TRBD2-1	TRBJ2-5	22	1.78E-04	0.00E+00	9.68E-06	2.75E-04	0.00E+00	3.78E-06	0.00E+00	0.00E+00	3.03E-06
ORF18	ILIEGIFFVS	TGTGCCAGCAGTGACTTGGTCGTTACTACTCCTACGAGCAGTACTTCG	CASSDLVVTTPTSSTS	TRBV6-4	TRBD2-1	TRBJ2-7	23	1.42E-04	4.10E-04	6.45E-06	1.72E-04	1.78E-04	1.89E-05	4.58E-04	3.19E-04	1.52E-05
ORF18	ILIEGIFFVS	TGTGCCAGCAGCCCCTCGACCTCCTACGAGCAGTACTTC	CASSPSTSYEQYF	TRBV9-1	TRBD1	TRBJ2-7	24	1.25E-04	1.37E-04	1.87E-03	3.44E-05	0.00E+00	1.84E-03	1.53E-04	0.00E+00	1.43E-03
ORF18	ILIEGIFFVS	TGTGCCAGCAGTGAATGGGGCACCAGGCGTGGCTACACCTTC	CASSEWGTRRGYTF	TRBV6-1	TRBD1-1	TRBJ1-2	25	1.25E-04	0.00E+00	3.10E-03	3.44E-05	0.00E+00	1.84E-03	0.00E+00	4.55E-05	2.54E-03
ORF18	ILIEGIFFVS	TGCGCCAGCAGCCAGACTAGCATACGGGATGAGCAGTTCTTC	CASSQTSIRDEQFF	TRBV4-1	TRBD2-1	TRBJ2-1	26	1.07E-04	0.00E+00	1.97E-04	6.88E-05	0.00E+00	1.29E-04	7.64E-05	1.37E-04	1.64E-04
ORF18	ILIEGIFFVS	TGTGCCAGCAGCTTAGCCCCAGGGGCAACTAATGAAAAACTGTTTTTT	CASSLAPGATNEKLFF	TRBV7-6	TRBD1-1	TRBJ1-4	27	1.07E-04	0.00E+00	2.28E-03	1.03E-04	8.88E-05	1.41E-03	0.00E+00	0.00E+00	1.78E-03
ORF18	ILIEGIFFVS	TGTGCCAGTAGTTTTACGTCCGGGGGCAATCAGCCCCAGCATTTT	CASSFTSGGNQPQHF	TRBV19-1	TRBD2-1	TRBJ1-5	28	1.07E-04	0.00E+00	1.00E-04	1.03E-04	8.88E-05	1.51E-05	7.64E-05	0.00E+00	1.21E-05
ORF18	ILIEGIFFVS	TGTGCCAGTAGTATGGGGGGGACCCAGTACTTC	CASSMGGTQYF	TRBV19-1	TRBD2-1	TRBJ2-5	29	8.90E-05	0.00E+00	3.23E-06	6.88E-05	8.88E-05	3.78E-06	0.00E+00	0.00E+00	0.00E+00
ORF18	ILIEGIFFVS	TGTGCCAGCAGCCCGGACAGTCTTTTACCACAGATACGCAGTATTTTG	CASSPDSLLPQIRSIL	TRBV28-1	TRBD1-1	TRBJ2-3	30	7.12E-05	0.00E+00	2.58E-05	1.72E-04	0.00E+00	3.03E-05	0.00E+00	0.00E+00	6.06E-05
ORF18	ILIEGIFFVS	TGTGCCAGCAGCGCCGGACAGGGAATCTACGAGCAGTACTTC	CASSAGQGIYEQYF	TRBV6-5	TRBD1-1	TRBJ2-7	31	7.12E-05	0.00E+00	3.23E-05	6.88E-05	0.00E+00	0.00E+00	0.00E+00	0.00E+00	2.43E-05
ORF18	ILIEGIFFVS	TGTGCCACCAGCGTTCCGTTCAGCTCCTACAATGAGCAGTTCTTC	CATSVPFSSYNEQFF	TRBV6-2	TRBD1-1	TRBJ2-1	32	7.12E-05	0.00E+00	1.29E-05	0.00E+00	0.00E+00	1.51E-05	0.00E+00	0.00E+00	2.12E-05
ORF18	ILIEGIFFVS	TGCAGTGCGAATACAGGATCCTACGAGCAGTACTTC	CSANTGSYEQYF	TRBV20	TRBD1-1	TRBJ2-7	33	5.34E-05	0.00E+00	6.55E-04	2.06E-04	8.88E-05	7.07E-04	7.64E-05	1.82E-04	7.16E-04
ORF18	ILIEGIFFVS	TGCGCCAGCAGTCCGCATTTTAGCGGGGCTTACAATGAGCAGTTCTTC	CASSPHFSGAYNEQFF	TRBV4-1	TRBD2-1	TRBJ2-1	34	5.34E-05	0.00E+00	3.07E-04	1.38E-04	0.00E+00	2.19E-04	0.00E+00	9.11E-05	4.55E-04
ORF18	ILIEGIFFVS	TGTGCCAGCAGCCAAGAAGGGACAGGGGGCATCGGGGAGCTGTTTTTT	CASSQEGTGGIGELFF	TRBV4-2	TRBD1-1	TRBJ2-2	35	5.34E-05	0.00E+00	1.68E-04	3.44E-05	0.00E+00	1.93E-04	1.53E-04	0.00E+00	2.30E-04
ORF18	ILIEGIFFVS	TGTGCCAGCAGTCACCCAGACAAAAAACTGTTTTTT	CASSHPDKKLFF	TRBV12-3/1	TRBD1-1	TRBJ1-4	36	5.34E-05	0.00E+00	4.84E-05	1.38E-04	0.00E+00	6.05E-05	0.00E+00	0.00E+00	1.33E-04
ORF18	ILIEGIFFVS	TGTGCCAGCACCTTTAGCGGGGAGAACACCGGGGAGCTGTTTTTT	CASTFSGENTGELFF	TRBV5-4	TRBD2-1	TRBJ2-2	37	5.34E-05	0.00E+00	0.00E+00	3.44E-05	0.00E+00	3.78E-06	0.00E+00	0.00E+00	9.10E-06
ORF18	ILIEGIFFVS	TGTGCCAGCAGACCTGGGACTAGCCCGAACACCGGGGAGCTGTTTTTT	CASRPGTSPNTGELFF	TRBV3-1/3-2	TRBD2-1	TRBJ2-2	38	3.56E-05	0.00E+00	4.84E-05	2.06E-04	1.78E-04	5.29E-05	0.00E+00	0.00E+00	9.10E-05
ORF18	ILIEGIFFVS	TGCAGTGCTAGGTCCGTCGCGAACACCGGGGAGCTGTTTTTT	CSARSVANTGELFF	TRBV20	TRBD2-1	TRBJ2-2	39	3.56E-05	2.73E-04	2.90E-05	3.44E-05	0.00E+00	1.51E-05	0.00E+00	0.00E+00	9.10E-06
ORF18	ILIEGIFFVS	TGTGCCAGCAGCCAAGAACAGGGAATGTCCAAAAACATTCAGTACTTC	CASSQEQGMSKNIQYF	TRBV4-2	TRBD1-1	TRBJ2-4	40	3.56E-05	0.00E+00	7.10E-05	6.88E-05	0.00E+00	3.03E-05	0.00E+00	0.00E+00	5.16E-05
ORF18	ILIEGIFFVS	TGTGCCAGCAGCTTAGACGGGACTAGCGGGGCTTACACAGATACGCAGTATTTT	CASSLDGTSGAYTDTQYF	TRBV7-6	TRBD2-1	TRBJ2-3	41	3.56E-05	0.00E+00	1.29E-05	6.88E-05	0.00E+00	1.51E-05	0.00E+00	0.00E+00	2.12E-05
ORF18	ILIEGIFFVS	TGTGCCAGCAGCTTGGAAGCCTCGGAGGTCCTGACTTTC	CASSLEASEVLTF	TRBV5-6	TRBD2-1	TRBJ2-6	42	3.56E-05	0.00E+00	9.68E-06	3.44E-05	0.00E+00	7.56E-06	0.00E+00	0.00E+00	3.03E-06
ORF18	ILIEGIFFVS	TGTGCCAGCAGGGAAATCGGGGTTCAAGAGACCCAGTACTTC	CASREIGVQETQYF	TRBV6	TRBD2-1	TRBJ2-5	43	3.56E-05	0.00E+00	0.00E+00	5.16E-04	0.00E+00	0.00E+00	9.17E-04	0.00E+00	0.00E+00
ORF18	ILIEGIFFVS	TGTGCCACCAGTGATCCTCCCCGGGACAGGGGCCCGGGGGAGCTGTTTTTT	CATSDPPRDRGPGELFF	TRBV24-1	TRBD1-1	TRBJ2-2	44	1.78E-05	1.37E-04	6.45E-06	2.06E-04	1.78E-04	3.78E-06	3.82E-04	3.19E-04	1.82E-05
ORF18	ILIEGIFFVS	TGTGCCAGCAGTTTGGGACAGGGGTACACTGAAGCTTTCTTT	CASSLGQGYTEAFF	TRBV12	TRBD1-1	TRBJ1-1	45	1.78E-05	0.00E+00	4.20E-05	3.44E-05	0.00E+00	3.03E-05	2.29E-04	9.11E-05	8.19E-05
ORF18	ILIEGIFFVS	TGCAGTGCCCTGGACGGGGACTCTGGAAACACCATATATTTT	CSALDGDSGNTIYF	TRBV20	TRBD2-1	TRBJ1-3	46	1.78E-05	0.00E+00	2.65E-04	1.38E-04	0.00E+00	1.74E-04	7.64E-05	0.00E+00	2.61E-04
ORF18	ILIEGIFFVS	TGCGCCAGCAGCCTGAGACCGGACGACAATGAGCAGTTCTTC	CASSLRPDDNEQFF	TRBV4-1	TRBD1	TRBJ2-1	47	1.78E-05	0.00E+00	1.19E-04	0.00E+00	0.00E+00	7.94E-05	7.64E-05	1.37E-04	1.06E-04
ORF18	ILIEGIFFVS	TGTGCCATCAGTGAGTCGACACCTAACTATGGCTACACCTTC	CAISESTPNYGYTF	TRBV10-3	TRBD1-1	TRBJ1-2	48	1.78E-05	0.00E+00	1.29E-05	1.03E-04	0.00E+00	1.13E-05	7.64E-05	0.00E+00	1.52E-05
ORF18	ILIEGIFFVS	TGCGCCAGCAGCTTGGTTAGCGGGAGAGACCCAGTACTT	CASSLVSGRDPVL	TRBV5-1	TRBD2-1	TRBJ2-5	49	1.78E-05	0.00E+00	1.29E-04	6.88E-05	0.00E+00	7.56E-05	0.00E+00	0.00E+00	9.40E-05
ORF18	ILIEGIFFVS	TGTGCTAGTGACCACTCTAGCGGGGTGATGGGTCAGATACGCAGTATTTTG	CASDHSSGVMGQIRSIL	TRBV12-5	TRBD2-1	TRBJ2-3	50	1.78E-05	0.00E+00	0.00E+00	3.44E-05	8.88E-05	0.00E+00	7.64E-05	9.11E-05	0.00E+00
ORF18	ILIEGIFFVS	TGCAGTGCTAGCAGGGATCGGGACAATGAGCAGTTCTTC	CSASRDRDNEQFF	TRBV20	TRBD1-1	TRBJ2-1	51	1.78E-05	0.00E+00	1.29E-05	3.44E-05	0.00E+00	3.78E-05	0.00E+00	0.00E+00	4.55E-05
ORF18	ILIEGIFFVS	TGTGCCACGACCGTACGAGGACGGGGTACGCAGTATTTT	CATTVRGRGTQYF	TRBV6-5	TRBD2-1	TRBJ2-3	52	1.78E-05	0.00E+00	6.45E-06	0.00E+00	0.00E+00	1.51E-05	7.64E-05	0.00E+00	9.10E-06
ORF18	ILIEGIFFVS	TGTGCCAGCAGTGTTTCCTACGAGCAGTACTTC	CASSVSYEQYF	TRBV6-2/6-3	.	TRBJ2-7	53	1.78E-05	0.00E+00	2.26E-05	3.44E-05	0.00E+00	3.03E-05	0.00E+00	0.00E+00	1.52E-05
ORF18	ILIEGIFFVS	TGTGCCAGCAGTTACGATAGGCCCCGACCCAGGGGCGGACAAGAGACCCAGTACTTC	CASSYDRPRPRGGQETQYF	TRBV6	TRBD1-1	TRBJ2-5	54	1.78E-05	0.00E+00	6.45E-06	0.00E+00	0.00E+00	1.51E-05	0.00E+00	0.00E+00	6.06E-06
ORF18	ILIEGIFFVS	TGCGCCAGCAGCCCCCAAGCTGCCTACGAGCAGTACTTC	CASSPQAAYEQYF	TRBV5-1	TRBD2-1	TRBJ2-7	55	1.78E-05	0.00E+00	9.68E-06	0.00E+00	0.00E+00	3.78E-06	0.00E+00	0.00E+00	3.03E-06
ORF18	ILIEGIFFVS	TGTGCCAGCAGCTTCTCAGGACAGGGGTATTCACCCCTCCACTTT	CASSFSGQGYSPLHF	TRBV11-2	TRBD1-1	TRBJ1-6	56	0.00E+00	0.00E+00	8.39E-05	3.44E-05	8.88E-05	6.43E-05	4.58E-04	2.28E-04	7.58E-05
ORF18	ILIEGIFFVS	TGCGCCAGCAGCTTGAGGGGGTCTAGCGGGGAGAGAGGCGATGAGCAGTTCTTC	CASSLRGSSGERGDEQFF	TRBV5-1	TRBD2-1	TRBJ2-1	57	0.00E+00	1.37E-04	6.45E-06	3.44E-05	8.88E-05	3.78E-06	0.00E+00	4.55E-05	3.03E-06
ORF18	ILIEGIFFVS	TGTGCCAGCAGCCCAAAAGGTATCGCTGAAGCTTTCTTT	CASSPKGIAEAFF	TRBV7-3	TRBD1	TRBJ1-1	58	0.00E+00	0.00E+00	1.99E-03	6.88E-05	0.00E+00	1.86E-03	0.00E+00	4.55E-05	2.46E-03
ORF18	ILIEGIFFVS	TGCGCCAGCAGCTGCAGGGAGATCCAAAAACATTCAGTACTTCGG	CASSCREIQKHSVLR	TRBV5-1	TRBD1	TRBJ2-4	59	0.00E+00	0.00E+00	3.23E-05	0.00E+00	8.88E-05	1.89E-05	2.29E-04	1.37E-04	1.52E-05
ORF18	ILIEGIFFVS	TGTGCCAGTTCTAGGGGGAACACTGAAGCTTTCTTT	CASSRGNTEAFF	TRBV19-1	TRBD1-1	TRBJ1-1	60	0.00E+00	2.73E-04	2.26E-05	3.44E-05	0.00E+00	7.56E-06	7.64E-05	0.00E+00	1.21E-05
ORF18	ILIEGIFFVS	TGTGCCAGCAGCTGGACAGGGGCCGCCAATTCACCCCTCCACTTT	CASSWTGAANSPLHF	TRBV2-1	TRBD1-1	TRBJ1-6	61	0.00E+00	0.00E+00	3.87E-05	0.00E+00	0.00E+00	7.19E-05	7.64E-05	4.55E-05	3.94E-05
ORF18	ILIEGIFFVS	TGCGCCAGCAGTGAGGGGACCCAGCCCCAGCATTTT	CASSEGTQPQHF	TRBV10-1	TRBD1	TRBJ1-5	62	0.00E+00	0.00E+00	2.58E-05	0.00E+00	0.00E+00	2.65E-05	7.64E-05	4.55E-05	2.12E-05
ORF18	ILIEGIFFVS	TGTGCCAGCAGCTTACGTCTGAACACTGAAGCTTTCTTT	CASSLRLNTEAFF	TRBV11-1	TRBD2-1	TRBJ1-1	63	0.00E+00	0.00E+00	2.26E-05	0.00E+00	0.00E+00	3.78E-06	1.53E-04	9.11E-05	6.06E-06
ORF18	ILIEGIFFVS	TGTGCCAGCAGCTTAGGCGGGGTCGATGGCTACACCTTC	CASSLGGVDGYTF	TRBV7-9	TRBD2-1	TRBJ1-2	64	0.00E+00	0.00E+00	1.94E-05	3.44E-05	0.00E+00	1.51E-05	0.00E+00	0.00E+00	3.03E-06
ORF18	ILIEGIFFVS	TGCGCCAGCAGCCAAGGGCCCCTGGGCGGTGAAGCTTTCTTT	CASSQGPLGGEAFF	TRBV4-1	TRBD1	TRBJ1-1	65	0.00E+00	0.00E+00	6.45E-06	1.03E-04	0.00E+00	3.78E-06	0.00E+00	0.00E+00	2.43E-05
ORF18	ILIEGIFFVS	TGTGCCAGCAGCCGCTTCAGGGACACCGGGGAGCTGTTTTTT	CASSRFRDTGELFF	TRBV5-8	TRBD1-1	TRBJ2-2	66	0.00E+00	0.00E+00	1.23E-04	0.00E+00	0.00E+00	1.29E-04	0.00E+00	0.00E+00	1.15E-04
ORF18	ILIEGIFFVS	TGTGCCAGCAGCCAAGAGGGGACAGGGGGCATCTACGAGCAGTACTTC	CASSQEGTGGIYEQYF	TRBV4-2	TRBD1-1	TRBJ2-7	67	0.00E+00	0.00E+00	2.26E-05	0.00E+00	0.00E+00	3.78E-06	0.00E+00	0.00E+00	4.25E-05
ORF18	ILIEGIFFVS	TGTGCCAGCAGCCTGGGGGGCGGGTCGGCTGGGGCCAACGTCCTGACTTTC	CASSLGGGSAGANVLTF	TRBV7-3	TRBD1	TRBJ2-6	68	0.00E+00	0.00E+00	1.61E-05	0.00E+00	0.00E+00	1.13E-05	0.00E+00	0.00E+00	2.73E-05
ORF53	FLYEGVLWL	TGTGCCAGCAGCAAACCTGAGGGGGCCAAAAACAATGAGCAGTTCTTC	CASSKPEGAKNNEQFF	TRBV27-1	TRBD1-1	TRBJ2-1	1	1.41E-03	0.00E+00	0.00E+00	5.16E-04	0.00E+00	0.00E+00	1.30E-03	0.00E+00	0.00E+00
ORF53	FLYEGVLWL	TGTGCCAGCAGCCAAGTTGCAGGCTACCCAGAGACCCAGTACTTC	CASSQVAGYPETQYF	TRBV3-1/3-2	TRBD1-1	TRBJ2-5	2	2.67E-04	5.47E-04	5.16E-05	5.85E-04	2.67E-04	3.03E-05	1.22E-03	4.10E-04	3.34E-05
ORF53	FLYEGVLWL	TGTGCCAGCAGCCTCCTCCTACAGGGCCACCAAGAGACCCAGTACTTC	CASSLLLQGHQETQYF	TRBV7-9	TRBD1-1	TRBJ2-5	3	3.56E-05	0.00E+00	0.00E+00	3.10E-04	0.00E+00	0.00E+00	0.00E+00	0.00E+00	0.00E+00
ORF53	FLYEGVLWL	TGTGCCAGCAGGACAGCGGGCGAGCAGTACTTC	CASRTAGEQYF	TRBV27-1	TRBD2-1	TRBJ2-7	4	1.78E-05	0.00E+00	0.00E+00	1.72E-04	0.00E+00	0.00E+00	2.07E-02	0.00E+00	0.00E+00
ORF62	ALWALPHAA	TGTGCCAGCAGTTACGCGGTCGGGCAACCGCGCAAGAGACCCAGTACTTC	CASSYAVGQPAQETQYF	TRBV6-2/6-3	TRBD1	TRBJ2-5	1	5.34E-05	0	0	1.72E-04	0	0	7.64E-05	0	0
ORF62	ALWALPHAA	TGTGCCAGCAGCCGACGGGGGGAAACAATTAGGGCACCCCTCCACTTT	CASSRRGETIRAPLHF	TRBV7	TRBD2-1	TRBJ1-6	2	0	0	0	0	8.88E-05	0	0	0	0
