# Supplementary material for: Selective retention of virus-specific tissue-resident T cells in healed skin after recovery from herpes zoster
Source: Nat Commun. 2022 Nov 15;13:6957. doi: 10.1038/s41467-022-34698-4 (PMC9663441; doi:10.1038/s41467-022-34698-4)
Supplement: Supplementary file 3 — Reporting Summary [file 41467_2022_34698_MOESM3_ESM.pdf]

nature portfolio

Corresponding author(s): Kerry J. Laing

Last updated by author(s): Oct 24, 2022

Reporting Summary

Nature Portfolio wishes to improve the reproducibility of the work that we publish. This form provides structure for consistency and transparency in reporting. For further information on Nature Portfolio policies, see our Editorial Policies and the Editorial Policy Checklist.

Statistics

For all statistical analyses, confirm that the following items are present in the figure legend, table legend, main text, or Methods section.

n/a	Confirmed	
☐	☒	The exact sample size (*n*) for each experimental group/condition, given as a discrete number and unit of measurement
☐	☒	A statement on whether measurements were taken from distinct samples or whether the same sample was measured repeatedly
☐	☒	The statistical test(s) used AND whether they are one- or two-sided<br>*Only common tests should be described solely by name; describe more complex techniques in the Methods section.*
☒	☐	A description of all covariates tested
☐	☒	A description of any assumptions or corrections, such as tests of normality and adjustment for multiple comparisons
☐	☒	A full description of the statistical parameters including central tendency (e.g. means) or other basic estimates (e.g. regression coefficient) AND variation (e.g. standard deviation) or associated estimates of uncertainty (e.g. confidence intervals)
☐	☒	For null hypothesis testing, the test statistic (e.g. *F*, *t*, *r*) with confidence intervals, effect sizes, degrees of freedom and *P* value noted<br>*Give P values as exact values whenever suitable.*
☒	☐	For Bayesian analysis, information on the choice of priors and Markov chain Monte Carlo settings
☒	☐	For hierarchical and complex designs, identification of the appropriate level for tests and full reporting of outcomes
☒	☐	Estimates of effect sizes (e.g. Cohen's *d*, Pearson's *r*), indicating how they were calculated

Our web collection on statistics for biologists contains articles on many of the points above.

Software and code

Policy information about availability of computer code

Data collection	TRB sequence data generated by Adaptive Biotech were exported from the Immunoseq platform. Flow cytometry data were collected on flow cytometers running BD FacsDiva Version 8.0.1. ELISA data were collected using Wallac 1420 Workstation software version 3 (PerkinElmer). Immunohistochemistry data were collected and analyzed using ZEN 2010 software (Version 6.0, Zeiss). Slides were scanned using a Zeiss Axio Imager II and images were analyzed using QuPath version 0.2.3. No custom code was or algorithms were used.
Data analysis	TRB sequences were analyzed using VDJTools (v1.2.1) and Immunarch (v0.6.6), using standard command lines detailed in the Methods. Flow cytometry data were analyzed using FlowJo (v10). Data management and manual filtering was performed using Microsoft Excel 2010. Statistical tests and graphs were generated using Prism (v8, GraphPad). No custom code was or algorithms were used. Relevant software are listed in the Methods section of manuscript where appropriate.

For manuscripts utilizing custom algorithms or software that are central to the research but not yet described in published literature, software must be made available to editors and reviewers. We strongly encourage code deposition in a community repository (e.g. GitHub). See the Nature Portfolio guidelines for submitting code & software for further information.

Data

Policy information about availability of data

All manuscripts must include a data availability statement. This statement should provide the following information, where applicable:

- Accession codes, unique identifiers, or web links for publicly available datasets
- A description of any restrictions on data availability
- For clinical datasets or third party data, please ensure that the statement adheres to our policy

Source data are provided with this paper, and in supplementary information files. TRB CDR3 sequences are available at ImmuneAccess: https://clients.adaptivebiotech.com/pub/laing-2022-nc and Zenodo (https://doi.org/10.5281/zenodo.7141050). Epitopes reported in Supplementary materials were submitted to the Immune Epitope Database (IEDB) and include Accession numbers, with raw data for protein and peptide level screens deposited at Zenodo (https://doi.org/10.5281/zenodo.7153680).

Human research participants

Policy information about studies involving human research participants and Sex and Gender in Research.

Reporting on sex and gender	Sex of study subjects is listed in Supplementary table 1, and stated in aggregate in the Results section (10 were male and 8 were female). We did not include sex-based analyses of our data because: 1) we have a small sample size, which limits these comparisons, and 2) there is no consistent association of herpes zoster with sex {see PMID 27382600}.
Population characteristics	From our Results section:<br>We recruited 19 persons with a clinical diagnosis of HZ. One was adjudicated to not have HZ based on the rash characteristics and was excluded. Amongst 18 persons with HZ (Supplementary Table 1), 10 were male and 8 were female, two of each were Black or Asian, one was multiracial, and 13 were white. Since age is important determinant of HZ risk, we included subjects across a wide range of ages: 3 subjects were under 30, 2 subjects were 30-39, 6 subjects were 40-49, 3 subjects were 50-59, and 4 subjects were over 60 years old. The median age was 47 years (range 21-71). HLA types were diverse; three persons had some degree of HLA-A or B homozygosity. None had immune suppressive conditions or chronic medications. Most HZ rashes involved the thoracic dermatome (N=13, 72%). Amongst 17 persons receiving oral antiviral therapy, 2 also received short-course oral corticosteroids. Three subjects self-reported receipt of a zoster vaccine: subject 81 (recombinant zoster vaccine, 48 months prior to HZ), subject 89 (unknown type and time), subject 91 (recombinant zoster vaccine, unknown time). Varicella vaccination status was not captured but most subjects were born before universal pediatric varicella vaccination began in the US in 1995. One person (subject 89, Supplementary Table 1) declined all biopsies, 6 subjects declined biopsies after day 90, and 11 completed the protocol to one year. One subject (subject 80) had an additional day 500 biopsy.
Recruitment	Subjects with HZ were recruited to University of Washington Virology Research Clinic approximately one month after HZ onset using the electronic health record query AMALGA system to identify participants. Younger individuals are potentially more likely to participate in clinical research, albeit, our case-finding method was unbiased regarding outreach to persons with a diagnosis of shingles in our health care system. Our median subject age of 47 is close the US-recommended age (50) for receiving the recombinant zoster vaccine: This age was set based on clinical trial data and the age of an upswing in HZ risk. During the recruitment interval (2018-2020) for this study, uptake for either shingles preventative vaccination in the US was quite low in eligible persons (recombinant zoster vaccine for people over 50; live attenuated zoster vaccine for people over 60). Therefore, we think it unlikely that our study was biased to enrolling younger persons due to vaccine-related low shingles rates in older people. We note a few participants in our study received shingles vaccination, but we are unable to assess whether this impacted the results. Study subjects were compensated per skin biopsy and for each blood draw. The study was approved by the University of Washington Institutional Review Board. Participants provided written informed consent.
Ethics oversight	The study was approved by the University of Washington Institutional Review Board.

Note that full information on the approval of the study protocol must also be provided in the manuscript.

Field-specific reporting

Please select the one below that is the best fit for your research. If you are not sure, read the appropriate sections before making your selection.

☒ Life sciences ☐ Behavioural & social sciences ☐ Ecological, evolutionary & environmental sciences

For a reference copy of the document with all sections, see nature.com/documents/nr-reporting-summary-flat.pdf

Life sciences study design

All studies must disclose on these points even when the disclosure is negative.

Sample size	No sample size calculation was performed. The number of persons with herpes zoster studied was chosen based on the ability of our clinic to enroll suitable persons willing to participate in a year-long research study. Because there were no preliminary data (in our lab or the literature)

	relevant to our assay endpoints available prior to the design our of research, it was not possible to conduct a power calculation in advance of starting the work. Due to the localized nature of herpes zoster, biopsy of clinically uninvolved, contralateral skin was appropriate to serve as control tissue. Therefore, paired statistical analyses could be used, as detailed in the manuscript. Two-sided statistical tests were used to preclude any a priori assumptions about the direction of differences between the two types of biopsies. The significance tests conducted were consistent with large magnitude differences for skin virus-specific T cell responses between the healed zoster and paired control skin tissues. The consistency of differences between zoster and control skin immune tests results (Fig. 3B, Fig. 3C, Fig. 4A, Fig. 4C), for several assays and for both CD4 and CD8 T cells, suggest that the sample size is sufficient.
Data exclusions	One study subject was excluded because the subject was adjudicated to not have herpes zoster based on the rash characteristics. Immunology data were not collected from this subject. No other data were excluded from analyses.
Replication	Replication was not applicable for several data types reported, including within sample ICS screening and TCR sequence analysis. ICS and TCR sequencing are single-cell assay such that hundreds to hundreds of thousands of individual cells are queried. Anti-VZV antibody ELISA and neutralization assays were performed with 2-fold titration of sera covering 6 concentrations, to improve robustness of measures. IFN- in sera of cultured T cells was measured ELISA assays were performed in duplicate or triplicate within assay, and at least 2 wells needed to exceed defined values to be considered positive. All T cell antigens reported to be reactive at the full-length open reading frame level were observed in within-assay duplicate or triplicate, and at least 2 wells needed to exceed defined values to be considered positive. All T cell epitopes reported were observed to be reactive in at least two separate assays performed on separate days, in addition to being detected in technical replicates within-assay.
Randomization	There was no randomization since this was not an interventional study and we were studying a time-course following disease symptom resolution in a small study cohort. Covariant analyses are irrelevant to our immunological study.
Blinding	Blinding was not relevant since was not an interventional study or clinical trial.

Reporting for specific materials, systems and methods

We require information from authors about some types of materials, experimental systems and methods used in many studies. Here, indicate whether each material, system or method listed is relevant to your study. If you are not sure if a list item applies to your research, read the appropriate section before selecting a response.

Materials & experimental systems

n/a	Involved in the study
☐	☒ Antibodies
☐	☒ Eukaryotic cell lines
☒	☐ Palaeontology and archaeology
☒	☐ Animals and other organisms
☒	☐ Clinical data
☒	☐ Dual use research of concern

Methods

n/a	Involved in the study
☒	☐ ChIP-seq
☐	☒ Flow cytometry
☒	☐ MRI-based neuroimaging

Antibodies

Antibodies used

anti-VZV gE (previously called gpI) Mab (Clone M1, Millipore MAB8612),
anti-CD3 (OKT3, Orthoclone, Ortho),
mAb supernatants from mouse hybridomas L243 (anti-DR), SPVL-3 (anti-DQ), B7/21 (anti-DP) (hybridoma source, ATCC),
anti-human CD28 (L293, BD 340975),
anti-human CD49d (L25, BD 340976),
anti-CD3-ECD (UCHT1, Beckman Coulter IM2705U),
anti-CD4–PE (A161A1; Biolegend 357404),
anti-CD4-FITC (S3.5; Invitrogen/Life Tech MHCD0401-4),
anti-CD8-FITC (3B5, Invitrogen/Life Tech MHCD0801-4),
anti-CD8-PerCP/Cy5.5 (SK1; BD 341051),
anti-IFN-g-PE/Cy7 (4S.B3; BD 557844),
anti-IL-2-APC (MQ1-17H12; BD 554567),
anti-CD4-BV421 (RPA-T4, BD 562424),
anti-CD3-APC/Cy7 (SP34-2, BD 557757),
anti-CD8 PE/Cy7 (RPA-T8, eBioscience 25-0088-42),
anti-CD4-PerCP (SK3, BD 566924),
anti-CD137-PE (4B4-1, Miltenyi 130-119-885),
mouse anti-CD4 (4B12; ThermoFisher MA5-12259),
mouse anti-CD8 (1A5; Monosan MONX10347),
mouse anti-CD8 (3B5, Thermo Fisher MHCD0800),
mouse anti-CD69 (FN50; Biolegend 310902),
mouse anti-CD103 (2G5-1; Thermo Fisher MA1-21434),
mouse anti-Ki67 (MIB-1; Dako GA62661-2),
mouse anti-PD-1 (EH33; Cell Signaling Technology),

mouse anti-TIA-1 (2G9A10F5; Beckman Coulter),
mouse anti-VZV IE63 (9D12; gift from Catherine Sadzot-Delvaux, University of Liege, Belgium, PMID: 11689044), available at https://doi.org/10.1006/viro.2001.1173
rabbit anti-CD3ε (SP7; Thermo Fisher, MA1-90582),
rat anti-CD8αβ (YTC182.20; Bio-Rad MCA351G),
rat anti-FoxP3 (PCH101; Thermo Fisher 14-4776-82).
Alexa Fluor 488 (AF488)-conjugated goat anti-mouse IgG1 (Thermofisher A-21121),
Alexa Fluor 488 (AF488)-conjugated goat anti-rat IgG (Thermofisher A-11006),
AF594-conjugated goat anti-mouse IgG2a (Thermofisher A-11012),
AF594-conjugated goat anti-rabbit IgG (Thermofisher A-21135),
AF647-conjugated chicken anti-rabbit IgG (Thermofisher A-21443),
AF647-conjugated goat anti-mouse IgG1 (Thermofisher A-21240),
goat anti-mouse IgG/ HRP conjugate (Invitrogen, G21040),
goat anti-human IgG-alkaline phosphatase conjugate (SeraCare, 5220-0351 (0751-1006))

Validation

anti-VZV gE, Per the vendor website: Recognizes the VZV glycoprotein I (now known as VZVgE)/ Also reacts with VZVgI (old name gpIV). Applications = immunofluorescence, immunoprecipitation, western blot, IHC. Validation: PMID: 21345964, PMID: 8151792, PMID: 7966575.
anti-CD3 (OKT3, Orthoclone, Ortho), Reactivity: human. Validated for expansion of T cells in many publications (e.g. PMID: 9883837).
mAb supernatants from mouse hybridomas L243 (anti-DR), SPVL-3 (anti-DQ), B7/21 (anti-DP) (hybridoma source, ATCC). Reactivity: human. Refer to PMID: 7512152 and PMID: 8169426. These mAbs were characterized in the 1980s as specific for all HLA-DR alleles, all HLA-DP alleles, or all HLA-DQ alleles.
anti-human CD28 (L293, BD 340975, RRID:AB_400197) and anti-human CD49d (L25, BD 340976), Reactivity: human. Validated for use as a co-stimulatory agent in intracellular cytokine staining PMID: 17451739. Per the vendor documentation: “When used in combination with CD49d (clone L25), CD28 (clone L293) provides optimal co-stimulatory signals for detection of antigen-specific cytokine production.”
anti-CD3-ECD (UCHT1, Beckman Coulter IM2705U, RRID:AB_130860), Reactivity: human (CD3epsilon). Application: Flow cytometry. Vendor validation statement: “UCHT1 reacts with the ε-chain of the CD3 complex. It was used as a CD3 reference mAb (ref.6T-CD3.1) during HLDA 6. After permeabilization with saponin-based permeabilization reagent IntraPrep, it may be used to detect intracytoplasmic CD3 by flow cytometry.”
anti-CD4–PE (A161A1; Biolegend 357404, RRID:AB_2562036). Reactivity: human. Application: flow cytometry. Per vendor: Each lot of this antibody is quality control tested by immunofluorescent staining with flow cytometric analysis.
anti-CD4-FITC (S3.5; Invitrogen/Life Tech MHCD0401-4, RRID: AB_2539719) Reactivity: human. Application: flow cytometry.
anti-CD8-FITC (3B5, Invitrogen/Life Tech MHCD0801-4, RRID:AB_2539725) Reactivity: human. Application: flow cytometry.
anti-CD8-PerCP/Cy5.5 (SK1; BD 341051, RRID:AB_400209) Reactivity: human. Application: flow cytometry.
anti-IFN-g-PE/Cy7 (4S.B3; BD 557844, RRID:AB_396894), Reactivity: Human (QC Testing), Rhesus, Cynomolgus, Baboon (Tested in Development) Application: Intracellular staining (flow cytometry) (Routinely Tested)
anti-IL-2-APC (MQ1-17H12; BD 554567, RRID:AB_398571), Reactivity: Human (QC Testing). Application: Intracellular staining (flow cytometry) (Routinely Tested).
anti-CD4-BV421 (RPA-T4, BD 562424, RRID:AB_11154417), Reactivity: Human (QC Testing). Application: Flow cytometry (Routinely Tested), Immunofluorescence (Reported).
anti-CD3-APC/Cy7 (SP34-2, BD 557757, RRID:AB_396863), Reactivity: Rhesus, Cynomolgus, Baboon (QC Testing), Human (Tested in Development). Application: Flow cytometry (Routinely Tested), Induction (Reported).
anti-CD8 PE/Cy7 (RPA-T8, eBioscience 25-0088-42, RRID:AB_1659702), Reactivity: Human, Non-human primate, Rat. Application: This RPA-T8 antibody has been reported for use in flow cytometric analysis.
anti-CD4-PerCP (SK3, BD 566924, RRID:AB_2739683), Reactivity: Human (QC Testing). Application: Flow cytometry (Routinely Tested)
anti-CD137-PE (4B4-1, Miltenyi 130-119-885, RRID:AB_2783944), Reactivity: human, non-human primate. Application: flow cytometry, immunofluorescence, Immunohistochemistry, mouse anti-CD4 (4B12; ThermoFisher MA5-12259),
mouse anti-CD8 (1A5; Monosan MONX10347), Reactivity: human CD8. Application: per vendor, “can be used for immunohistochemistry on frozen (acetone fixation recommended) and paraffin sections. It can also be used for Western Blotting.”
mouse anti-CD8 (3B5, Thermo Fisher MHCD0800, RRID:AB_10372813), Reactivity: Human, Mouse, Non-human primate, Rhesus monkey. Application: Immunocytochemistry (ICC/IF), Flow Cytometry (Flow), Immunoprecipitation (IP), ChIP assay (ChIP).
mouse anti-CD69 (FN50; Biolegend 310902, RRID:AB_314837), Reactivity: Human, African Green, Baboon, Chimpanzee, Cynomolgus, Pigtailed Macaque, Rhesus. Application: flow cytometry (QC tested), CyTOF (verified), IHC-F (reported in literature).
mouse anti-CD103 (2G5-1; Thermo Fisher MA1-21434, RRID:AB_558012), Reactivity: Cynomolgus monkey, Human, Non-human primate, Rhesus monkey. Application: Flow cytometry (1:50-1:100), Immunoprecipitation (Assay-dependent), IHC (F) (1:50-1:100).
mouse anti-Ki67 (MIB-1; Dako GA62661-2, RRID:AB_2687921), Reactivity: human. Application: Immunohistochemistry.
mouse anti-PD-1 (EH33; Cell Signaling Technology #43248, RRID:AB_2728836), Reactivity: human. Application: Immunohistochemistry.
mouse anti-TIA-1 (2G9A10F5; Beckman Coulter, IM2550, RRID:AB_131704), Reactivity: human. Application: intracellular flow cytometry, IHC. From vendor site: “The 2G9 antibody is a useful reagent for detecting cytolytic effector cells in tissue infiltrates. It was evaluated during HLDA 5 in the section of monoclonal antibodies reactive with intracellular antigens. It may be used to detect intracytoplasmic TIA-1 in flow cytometry after permeabilization with IntraPrep permeabilization reagent.” IHC application was independently validated PMID: 28817800.
mouse anti-VZV IE63 (9D12; gift from Catherine Sadzot-Delvaux, University of Liege, Belgium), Reactivity to IE63 protein of varicella zoster virus. Validation: PMID: 11689044. “can be used for EIA, immunohistochemistry, or Western blotting analysis. In immunohistochemistry this monoclonal anti-body recognizes the protein in cell cultures, frozen sections fixed with acetone:ethanol at a ratio of 1:1, or paraffin-embedded tissues.”
rabbit anti-CD3ε (SP7; Thermo Fisher, MA1-90582, RRID:AB_1956722), Reactivity: Baboon, Dog, Human, Mouse, Non-human primate, Pig, Rat, Rhesus monkey. Application: Flow cytometry (1:1,000), Immunohistochemistry (IHC), IHC (Paraffin) (1:150), IHC (Frozen) (1:500), Western Blot (1:200), Immunocytochemistry (ICC/IF).
rat anti-CD8αβ (YTC182.20; Bio-Rad MCA351G, RRID:AB_877504). Reactivity human, simian, rhesus monkey, cynomolgus monkey.

Application: Flow Cytometry; Immunohistochemistry; Immunohistology – Frozen.
rat anti-FoxP3 (PCH101; Thermo Fisher 14-4776-82, RRID:AB_467554). Reactivity: chimpanzee, cynomolgus monkey, human, non-human primate, rhesus monkey. Application: Western Blot (1-5 µg/mL), IHC (F) (10 µg/mL), IHC (P) (10 µg/mL), ChIP assay (ChIP), Flow Cytometry (Flow).
Alexa Fluor 488 (AF488)-conjugated goat anti-mouse IgG1, polyclonal (Thermofisher A-21121, RRID:AB_2535764). Reactivity: mouse. Application: IHC (F) (1:2,000), Flow cytometry (1-10 µg/mL), ICC/IF (1 µg/mL)
Alexa Fluor 488 (AF488)-conjugated goat anti-rat IgG, polyclonal (Thermofisher A-11006, RRID:AB_2534074). Reactivity: rat. Application: ICC/IF (4 µg/mL), Flow cytometry (1-10 µg/mL)
AF594-conjugated goat anti-rabbit IgG, polyclonal (Thermofisher A-11012, RRID: AB_2534079). Reactivity: rabbit. Applications: Immunocytochemistry (ICC/IF)(2µg/mL), Flow Cytometry (1-10 µg/mL).
AF594-conjugated goat anti-mouse IgG2a, polyclonal (Thermofisher A-21135 RRID:AB_2535774) Reactivity: mouse. Applications: Flow (1-10 µg/mL), IHC (1-10 µg/mL), ICC/IF (1 µg/mL)
AF647-conjugated chicken anti-rabbit IgG, polyclonal (Thermofisher A-21443, RRID: AB_2535861). Reactivity: rabbit. Applications: Immunohistochemistry (IHC) (1-10 µg/mL), Immunocytochemistry (ICC/IF) (4 µg/mL)
AF647-conjugated goat anti-mouse IgG1, polyclonal (Thermofisher A-21240 RRID:AB_2535809). Reactivity: mouse. Applications: Immunohistochemistry (IHC)(1-10 µg/mL), Immunocytochemistry (ICC/IF)(1-10 µg/mL).
goat anti-mouse IgG/ HRP conjugate, polyclonal (Invitrogen, G21040 RRID:AB_2536527). Reactivity: mouse. Application: Western Blot (WB) 1:10,000-1:200,000; Immunohistochemistry (IHC) 1:500-1:2,000; ELISA (ELISA) 1:500-1:2,000.
goat anti-human IgG-alkaline phosphatase conjugate, polyclonal (SeraCare, 5220-0351 (0751-1006)) Reactivity: Human. Per vendor, "This product reacts specifically with human IgG and may recognize other immunoglobulin types that have light chains in common with IgG. Reactivity to IgG subclasses has not been tested. Antibodies to human IgG may cross react with immunoglobulins of other mammalian species if common bonding sites are shared." Application: Per vendor, "Tested by gel diffusion and ELISA techniques".

No newly developed antibodies were used.

Eukaryotic cell lines

Policy information about cell lines and Sex and Gender in Research

Cell line source(s)	COS-7 cells (ATCC CRL-1651, C. aethiops origin, RRID:CVCL_0224) used for making artificial antigen presenting cells that express HLA class I.<br>RM3 (human male B cell lymphoma cell origin, derivative of Raji cell (ATCC CCL-86) generated by Calman and Peterlin, University of California, PMID: 3116083) and DAP.3 (mouse fibroblast origin, RRID:CVCL_V055, derivative of (L-M(TK-), PMID: 6952248), ATCC CCL-1.3)-based single antigen lines (e.g. expressing single HLA class II; PMID: 23392739) and HLA negative control lines were obtained from Alex Sette (La Jolla Institute).<br>MRC-5 cells (ATCC CCL-171, human fibroblasts from lung tissue male, 14-week-old embryo, RRID:CVCL_0440)<br>Primary human lung fibroblasts (HLF, female origin, ATCC Cat # CCL-199, RRID:CVCL_2255).<br>Demographics, including sex, for human primary T cell lines generated during this study are listed in Supplementary Table 1.
Authentication	No cell lines were genotypically authenticated. The RM3 and DAP.3-based cell lines expressing single HLA class II were validated as expressing either HLA DR, HLA DP, or HLA DQ as appropriate by staining them with the appropriate mAb and flow cytometry.
Mycoplasma contamination	Routine mycoplasma testing was performed with the Lonza MycoAlert Mycoplasma Detection Kit. Only Mycoplasma negative cell lines were used.
Commonly misidentified lines<br>(See ICLAC register)	No commonly misidentified lines were used.

Flow Cytometry

Plots

Confirm that:

- [x] The axis labels state the marker and fluorochrome used (e.g. CD4-FITC).
- [x] The axis scales are clearly visible. Include numbers along axes only for bottom left plot of group (a 'group' is an analysis of identical markers).
- [x] All plots are contour plots with outliers or pseudocolor plots.
- [x] A numerical value for number of cells or percentage (with statistics) is provided.

Methodology

Sample preparation	PBMC were prepared from venous anticoagulated blood by FIcoll Hypaque density gradient centrifugation and stored in liquid nitrogen prior to use. Viability was assessed by trypan blue staining after thaw and all functional assays of PBMC included positive controls to ensure cell functional reactivity. Skin T cells were obtained, in brief, from punch biopsies which were minced and subjected to initial outgrowth using a non specific mitogen (PHA), a cytokine preparation containing IL-2, and feeder cells.<br><br>VZV-specific CD4 T cells were quantified in PBMC by intracellular cytokine staining (ICS) flow cytometry. After overnight rest in TCM, 10^6 PBMC were incubated for 18 hours at 37oC with costimulatory antibodies (anti-CD28 and anti-CD49d; BD

Biosciences) and UV-VZV or UV-mock (1:100), medium, or PHA-P (1.6 µg/mL) in TCM: Brefeldin-A (Sigma) was added after 2 hours. After Live/Dead Fixable Near-IR (Invitrogen) stain, cells were treated with FACS Lysing and Permeabilizing 2 solutions (BD) and stained with anti-CD3-ECD (UCHT1, Beckman Coulter), anti-CD4–PE (A161A1; Biolegend) or anti-CD4-FITC (S3.5; Invitrogen), anti-CD8-FITC (3B5, Invitrogen) or anti-CD8-PerCP/Cy5.5 (SK1; BD), anti-IFN-g-PE/Cy7 (4S.B3; BD), and anti-IL-2-APC (MQ1-17H12; BD).

Skin CD4 T cells reactive to whole VZV was initially assessed using ICS. Biopsy-derived, PHA-expanded T cells (2.5 × 10^5) were combined with equal numbers of cell tracker violet (CTV, Invitrogen)–stained autologous PBMC and antigens described for ex vivo assays. Cell permeabilization and antibody staining was also the same as for ex vivo assays.

For selected epitopes, PE- or allophycocyanin-conjugated HLA class I tetramers were constructed (Flex-T, Biolegend, per manufacturer). Tetramers were used to stain T cells (2 x 10^6) for 30 min on ice in the dark, followed by staining with antiCD3-ECD (UCHT1, Beckman Coulter), antiCD4-BV421 (RPA-T4, BD), antiCD8-FITC (3B5; Thermofisher), and 7-AAD (BD).

Instrument

ICS analyses were performed on a BD LSRII cytometer. Cell sorting was performed on a BD Aria II.

Software

Event acquistion was performed using FACS Diva. Data analysis was completed using FlowJo version 10 (for Mac).

Cell population abundance

Due to the low cell numbers obtained when sorting tetramer-specific populations, we did not confirm purity levels of the sorted fractions by flow cytometry. Sorted populations of T cells were only used for determining TCR sequences of tetramer-specific T cells.

Gating strategy

Gating strategies are shown in Supplementary Figure 5.

A) Ex vivo abundance of cytokine (IFN-γ and/or IL-2) positive CD4 T cells in PBMC exposed to VZV or control antigens: Single live CD3+CD4+CD8– lymphocytes were assessed for IFN- and IL-2. Lymphocytes were selected based on forward (FSC-A) and side (SSC-A) scatter characteristics. Doublets/aggregates were removed, and single cells selected, based on spreading of events when FSC-H was plotted against FSC-A. Live CD3+ T cells were selected as LiveDead negative cells that were CD3 positive, from which CD4 positive, CD8 negative events were gated. From the CD4+CD8- gate, IL-2 was gated against IFN-, and all IFN-γ and/or IL-2 positive events were considered “cytokine positive”.

B) VZV-specific CD4 T cells in skin biopsy-derived T cell lines when CTV-stained PBMC are used as antigen-presenting cells. Single live (CTV dump-gated) CD3+CD4+CD8– lymphocytes were assessed for IFN- and IL-2. Lymphocytes were selected based on forward (FSC-A) and side (SSC-A) scatter characteristics. Doublets/aggregates were removed, and single cells selected, based on spreading of events when FSC-H was plotted against FSC-A. LiveDead positive (dead) and CTV positive (added PBMC) were dump-gated, and CD3 positive cells (vs SSC-A) were selected from events negative for both of these markers. CD4 positive, CD8 negative events were separated from the gated CD3 positive cells. From the CD4+CD8- gate, IL-2 was gated against IFN-, and all IFN-γ and/or IL-2 positive events were considered “cytokine positive”.

C) Sorting tetramer-specific CD8 T cells from skin biopsy-derived T cell lines. Example shows subject 79, day 360 rash site T cell line. Single live CD3+CD4-CD8+ lymphocytes that bound VZV-specific tetramers were sorted. Lymphocytes were selected based on forward (FSC-A) and side (SSC-A) scatter characteristics. Doublets/aggregates were stringently removed, and single cells selected, based on spreading of events when SSC-H vs SSC-W followed by FSC-H vs FSC-W. Live CD3+ T cells were selected as 7-AAD negative cells that were CD3 positive, from which CD8 positive, CD4 negative events were gated. From the CD8 positive T cell gate, PE-conjugated and APC-conjugated tetramers were visualized and gated for sorting.

☒ Tick this box to confirm that a figure exemplifying the gating strategy is provided in the Supplementary Information.
